# Whole-family programmes for families living with parental mental illness: a systematic review and meta-analysis

**DOI:** 10.1007/s00787-024-02380-3

**Published:** 2024-02-23

**Authors:** B. Moltrecht, Aurelie M. C. Lange, H. Merrick, J. Radley

**Affiliations:** 1 Evidence-Based Practice Unit, Anna Freud Centre, University College London, London, UK; 2 Centre for Longitudinal Studies, University College London, London, UK; 3 Centre for Applied Research on Social Sciences and Law, Amsterdam University of Applied Sciences, Amsterdam, The Netherlands; 4 Population Health Sciences Institute, Newcastle University, Newcastle Upon Tyne, UK; 5 King’s College London, Institute of Psychiatry, Psychology and Neuroscience, London, UK

**Keywords:** Parental mental illness, Family mental health, Mental health intervention, Mental health prevention, Systematic review, Family programme

## Abstract

Several interventions have been developed to support families living with parental mental illness (PMI). Recent evidence suggests that programmes with whole-family components may have greater positive effects for families, thereby also reducing costs to health and social care systems. This review aimed to identify whole-family interventions, their common characteristics, effectiveness and acceptability. A systematic review was conducted according to PRISMA 2020 guidelines. A literature search was conducted in ASSIA, CINAHL, Embase, Medline, and PsycINFO in January 2021 and updated in August 2022. We double screened 3914 abstracts and 212 papers according to pre-set inclusion and exclusion criteria. The Mixed Methods Appraisal Tool was used for quality assessment. Quantitative and qualitative data were extracted and synthesised. Randomised-control trial data on child and parent mental health outcomes were analysed separately in random-effects meta-analyses. The protocol, extracted data, and meta-data are accessible via the Open Science Framework (https://osf.io/9uxgp/). Data from 66 reports—based on 41 independent studies and referring to 30 different interventions—were included. Findings indicated small intervention effects for all outcomes including children’s and parents’ mental health (*d*_c_ = −0.017, −027; *d*_p_ = −0.14, −0.16) and family outcomes. Qualitative evidence suggested that most families experienced whole-family interventions as positive, highlighting specific components as helpful, including whole-family components, speaking about mental illness, and the benefits of group settings. Our findings highlight the lack of high-quality studies. The present review fills an important gap in the literature by summarising the evidence for whole-family interventions. There is a lack of robust evidence coupled with a great need in families affected by PMI which could be addressed by whole-family interventions. We recommend the involvement of families in the further development of these interventions and their evaluation.

**Supplementary Information:**

The online version contains supplementary material available at 10.1007/s00787-024-02380-3.

## Background

Parental mental illness (PMI) negatively affects the life and mental health of all family members. Children of parents with mental illness are at increased risk of developing mental health difficulties as well as interpersonal, academic, and social difficulties in comparison to children growing up with parents who do not experience mental health difficulties. Furthermore, it has been reported that families with parental mental illness are more likely to experience social exclusion and are less likely to seek help. Due to this need, several psychosocial intervention programmes have been developed and implemented across different settings to support families (mostly parents and their children) living with parental mental illness [1, 2].

Most studies and reviews investigating the impact of interventions for families affected by PMI have focused on parent-only or child-only interventions [3]. However, recent evidence suggests that programmes with a family component, where both children and parents/carers receive support, may have greater impact as they benefit all family members and can therefore reduce costs to health and social care systems [2]. Qualitative research with parents has also highlighted that parents value whole-family approaches [4]. A few systematic reviews investigating programmes to support families with PMI have been conducted in recent years, reporting small effects of treating and preventing the development of mental illness in children [1, 2, 5]. The majority of systematic reviews [1–3, 5–7] primarily reported on child mental health outcomes and neglected to investigate a more comprehensive picture by also looking at outcomes relating to parental mental health and family functioning. Additionally, none of the recent reviews have investigated or reflected on the available evidence in relation to how families experience these whole-family interventions [7]. Furthermore, a recent systematic review of reviews investigating PMI interventions, highlighted that most studies so far had focused on mothers, and in particular the perinatal period [3].

Based on the above, the present review focuses on whole-family interventions that include at least parents/carers and their children. We aim to provide an overview of the interventions available for families affected by PMI, their characteristics and components, and the existing evidence around the interventions’ effectiveness in improving child and parent mental health outcomes as well as family outcomes. Additionally, we will investigate how families have experienced the interventions.

We answer the following research questions:

1. What types of whole-family interventions are available for families living with parental mental illness?
2. What are the core components of these whole-family interventions?
3. What is the evidence base for existing whole-family interventions and their effectiveness in enhancing child and parent mental health outcomes, and family outcomes?
4. How have families experienced taking part in whole-family interventions?

## Methods

The systematic review is reported in line with the PRISMA 2020 [8]. All review documents and data are accessible via the project page on the Open Science Framework [9].

### Search strategy and selection criteria

A literature search was conducted in ASSIA, CINAHL, Embase, Medline, and PsycINFO on 28th of January 2021, and an updated search was conducted in the same databases on the 3rd of August 2022 (see Supplement material ‘Search strategy’ for details). Identified records were exported into the Rayyan systematic review software [10]. References of relevant literature reviews were further screened for additional publications and manually added. Reports identified during the full-text screening, referring to the same study, were also added retrospectively. Our literature search focused primarily on reports published in peer-reviewed journals; however, we also screened the preprint server PsyArXiv and Google Scholar for studies and reports published elsewhere. Furthermore, we asked third sector organisations in our networks (i.e., Anna Freud National Centre and the Mental Health Foundation) to share relevant reports.

All abstracts and titles were screened (double-blind) by at least two researchers (HM, BM, KJ, JR, and AL). Four researchers conducted a pilot by screening the same ten reports against the selection criteria which were subsequently discussed with the team to clarify uncertainties before commencing the rest of the screening (for details and notes on adjustments and agreements made during the pilot screening, see [9]). Authors initially reported disagreements in 2.6% of the cases. The research team discussed the relevant papers and their eligibility until an agreement was found. Following this, the research team conducted full-text screenings of all remaining reports, with an initial 4% of disagreement amongst raters, which were then resolved.

We used the following criteria to screen and select studies:

#### Inclusion criteria

- Families where a parent had been clinically diagnosed with one or more mental illnesses, including substance abuse.
- Children’s age (sample mean) at least 5 years and younger than 24 years. Age of at least 5 years was chosen as this is the age where most postnatal or parent-supporting interventions stop (e.g., health visitors). Most interventions that focus on children younger than age 5 focus on postnatal mental illness and the mother–child relationship.
- Psychosocial interventions involving the whole family (at least one parent and one child were involved in at least one element of the programme, either separately or together).
- Psychosocial intervention designed to support families with parental mental illness.
- Reporting results for child or parent mental health outcomes, family functioning, and/or families’ experiences with whole-family interventions.
- Studies published in English, German or Dutch.

#### Exclusion criteria

- Interventions were child mental or physical illness was the only referral reason or the only focus of the intervention.
- Families affected by rare or specific medical or neurological conditions or exposed to traumatic events (e.g., cancer, traumatic brain injury, physical or cognitive disabilities, and environmental catastrophes).
- Studies including families affected by poverty, abuse, or violence but not reporting on parental mental health.
- Studies focussing on postnatal mental illness, with children younger than 5 years.
- Interventions including only parents or only children.
- Interventions focussing on medication, supplements, or changing specific aspects of a healthy lifestyle (e.g., diet, sleep, and physical exercise).
- Reports reporting on service model evaluations, case studies or reviews.
- Studies only reporting physiological test and medical examination outcomes, e.g., blood, genes, and MRI.

### Data extraction

Data were extracted and cross-checked by at least two researchers. We extracted the following data from each study: authors; country; year of publication; study design; intervention setting; outcome measures; intervention name; intervention type (such as multi-group or single family); intervention aim; presence of an intervention manual; intervention components; intervention structure, including number of sessions, length and frequency; measurements used and assessment time-points; sample characteristics including age, ethnicity, gender, diagnosis of parent; type and format of control group; summary statistics; results and interpretation by authors; and information on study quality.

### Identification and grouping of intervention components

We grouped and conceptualised the components for each intervention by screening and extracting the information from each included study. Subsequently, we coded the listed components and grouped them into their smallest meaningful unit. We compared these codes across studies and refined them further in discussions with the research team. The final components’ list was used to create a codebook of intervention components. Two authors (AML and JR) trialled the codebook for five studies and discussed any disagreements with the team which led to further refinements of the codebook. We categorised the final list of codes into higher level components, which were discussed and agreed with the whole team. The final codebook consisted of 22 components, grouped into five higher level components. The same two authors (AML and JR) coded all remaining studies and compared disagreements. When a consensus could not be made, a third author (HM) made the final decision.

### Quality assessment

The Mixed Methods Appraisal Tool (MMAT) was used to assess the methodological quality of the included studies [11]. The MMAT includes two questions that are used for all studies and then a subset of questions specific to the study design of the study. All questions were rated as “no”, “unclear”, or “yes”. The quality assessment was done by two authors (HM and AML). Each paper was individually assessed by each author, and then, differences in quality ratings were discussed and agreed. Agreement was reached on all quality ratings. We included all studies in this review, regardless of their quality rating, but reflect on the evidence in light of the methodological quality of the respective studies.

### Synthesis of available evidence

In some cases, multiple reports (*n*) were published for the same study (*t*); hence, we grouped the available evidence by study and subsequently also by intervention. We created an overview of all quantitative outcomes reported in the studies and summarised the evidence in three main categories: “parent mental health outcomes”, “child mental health outcomes”, and “family outcomes”.

### Meta-analyses

For the meta-analyses, we only used data from peer-reviewed publications as these are assumed to be of higher research quality. We excluded data from feasibility, pilot and acceptability studies and included data from randomised and non-randomised-controlled trials if they reported sufficient data on our outcomes of interest. We were unable to conduct a multi-level meta-analysis as most papers did not report correlations between measures or time-points. We conducted multiple random-effects meta-analyses instead. Only four studies reported data on incidences rates or risk ratios for different child outcomes (e.g., anxiety, depression, psychiatric status, suicide ideation, and substance use); hence, we did not conduct a meta-analysis to report on changes in risk or incidence following preventative treatments. Treatment effects in terms of symptom reduction were estimated using weighted mean effect size Hedges’ *g*, calculated with the “meta” command in Stata 16/17 [12], which requires post-intervention means, standard deviations, and sample sizes for both groups (see Formula 1). We clustered the outcome data into short-term (1:0 months to less than 6 months), medium–short term (2:6 months to less than 10 months), medium long-term (3: 10 months to less than 18 months), and long-term (4: 18 months and more) follow-up outcomes and ran separate meta-analyses by length of follow-up. We estimated effect sizes for parent and child outcomes separately. For child mental health outcomes, we distinguished between parent-reported versus child self-reported outcomes. A meta-analysis for family outcomes was not possible, because only four studies assessed any type of family outcome referring to different concepts and using different measures [i.e., parent behaviour (*t* = 4), parent–child relationship (*t* = 2), and sibling relationship (*t* = 1)].

Formula Hedges’ *g*

$$g = \frac{M1 - M2}{{\text{spooled }}}\;{\text{with}}\,{\text{spooled}}\;\sqrt {\frac{{(n1 - 1)s1^{2} + (n2 - 1)s2^{2} }}{n1 + n2 - 2}} .$$

We conducted sensitivity analyses for studies using multiple measures for similar outcomes (e.g., anxiety and internalising symptoms), by running separate meta-analyses with different outcome sets. Ideally, this is done in a multi-level meta-analysis involving all outcomes, but correlations between outcome measures were not reported.

Heterogeneity was investigated via *Q*-statistic, and *I*^2^ and *T*^2^ statistics. The *Q*-statistic estimates the probability of sampling error being the only cause for variance, while *T*^2^ describes between-study variance and *I*^2^ what proportion of the observed variance is due to systematic differences between the studies. Furthermore, each study’s level of heterogeneity was assessed using a Galbraith plot (“meta galbraithplot” command in Stata). Meta-regressions and subgroup analyses were used to investigate sources of bias and heterogeneity due to study-level factors, including type of control group (passive vs active), quality ratings, and number of intervention sessions. If any of the potential moderating factors were significant, further subgroup analyses were conducted. Publication bias was visually assessed using a funnel plot.

### Qualitative synthesis

Outcomes relating to families’ experiences with intervention and their acceptability were mainly reported in qualitative studies. For the qualitative synthesis, we extracted and analysed all qualitative result sections using thematic analysis with a realist approach following guidelines for thematic analysis [13, 14] and qualitative thematic synthesis by Thomas and colleagues [15]. Authors of the reviewed reports occasionally adopted different stances (e.g., constructivist [16]) which may have influenced the presentation of their results. Two authors (AML and BM) independently read, extracted, and coded the result sections, including quotations. Following this the two authors discussed their coding scheme to develop common themes. There were no substantial disagreements, but remaining uncertainties were presented and discussed with a third author (HM) to reach the final list of themes. The three authors (BM, AML, and HM) are female, of similar age and background and have both been involved in an evaluation study for a whole-family intervention for families affected by PMI at the time this review was undertaken. This may have influenced the high overlap and agreement in developing the themes.

Some studies assessed acceptability levels using questionnaires to capture intervention satisfaction or by reporting engagement and attendance rates. Where available we reported the quantitative evidence along with the qualitative findings.

## Results

### Study selection

We identified a total of 66 reports (*n*) that related to 41 individual studies (*t*) and evaluated 30 different interventions (*i*). We included 12 RCTs in the meta-analysis, 36 studies for the quantitative synthesis, and 22 reports to investigate families’ experiences with interventions, of which 10 reports provided qualitative data and 14 reports quantitative data regarding acceptability, satisfaction, and usefulness of the interventions. The flow diagram demonstrates the study selection process and reasons for excluding certain studies.
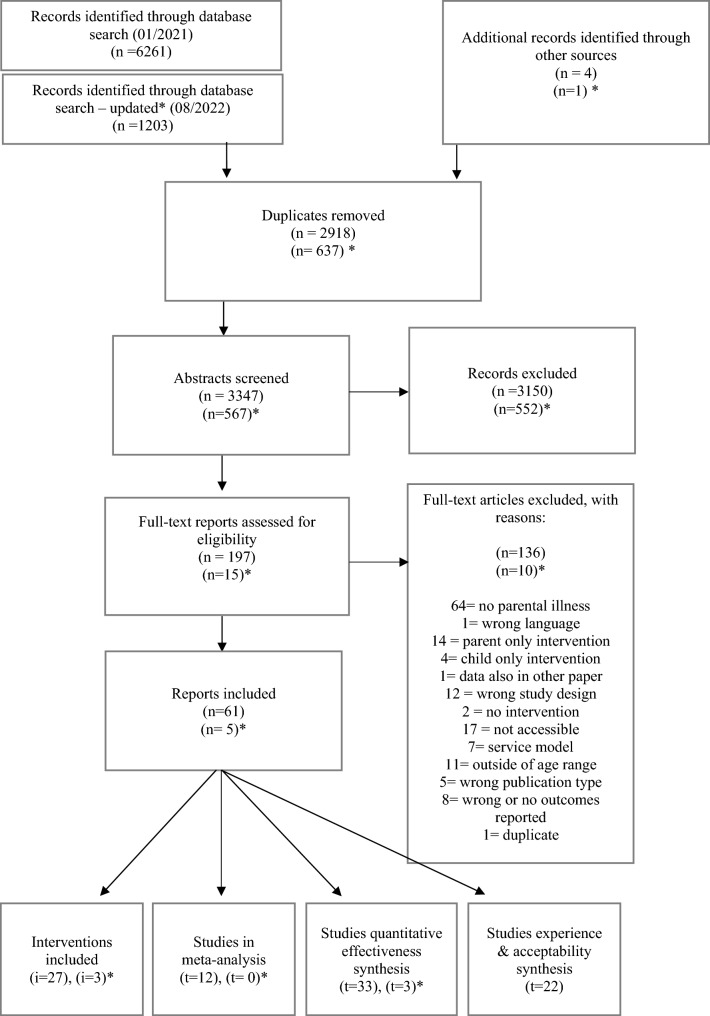


### Study characteristics and quality ratings

Most studies were conducted in the United States (51%; 21/41), five in the United Kingdom (12%; 5/41), four in Germany (9%; 4/41), and the rest in Finland, Australia, Greece, Ireland, Sweden, Canada, Spain, China, and Iran. Children’s ages in the study samples ranged from 3 to 19 years (mean age of 11.4 years). More details about sample characteristics per study, including age, gender, and ethnicity of parents and children, are provided in Table S1 in the supplements. Twenty-seven studies included a control group (65%; 27/41), of which 18 were RCTs, five non-randomised control trials and four feasibility or pilot trials. Eight studies (19%; 8/41) had a single-group design providing quantitative descriptive statistics, and ten studies (24%; 10/41) involved qualitative research methods (two of which were part of an RCT).

All reports were independently assessed by two researchers (HM and AML) using the MMAT. A total of 1491 ratings were made by each assessor, and in 68 cases (4%), further discussions and control checks were needed to find an agreement (the MMAT ratings sheet and cases that required more discussion is provided in the supplements). Over one-third of the papers (38%; 26/66) had a high-quality rating of four or five stars (80–100% of the items scored as ‘yes’), 28% (19/66) were given a medium-quality rating of three stars (60% quality score), and 28% (19/66) had a low rating of one or two stars (20–40% quality score). Two papers were of very low quality (i.e., no stars). A high-quality rating of 80–100% was received by 44% of the RCTs (8/18), 50% of the qualitative and mixed method papers (2/4), and 20% of the non-randomised trials (1/5) and 66% (4/6) of the descriptive quantitative studies. Table 1 presents an overview of the study characteristics.

**Table 1:** Overview of included trials and studies

Study ID	Report ID	Country	*N* families	Age of children	Control condition	Research design	Quality rating
1	1	Greece	*N*_t_ 30 N_c_ 32	_t_ 11.7 (2.6) _c_ 12.3 (2.7)	Active	RCT^a^	**
2	73	GER	*N*_t_ 28 *N*_c_ 49	M(SD) = 10.41 (2.66)	Passive	fRCT	**
3	93 119 123	Finland	*N*_t_ 53 *N*_c_ 56	11.9 (2.6)	Active	RCT^a^ RCT RCT	*** *** ****
4	186	USA	*N*_t_ 7	10.9 (2.0)	None	QUAND	***
5	180	USA	*N*_t+c_ 28	10.5 ( −)	Active	RCT	**
6	148 162 177 178 179	USA	*N*_t_ 55 *N*_c_ 44	11.6 (1.9) 11.5 (2.03)	Active	RCT RCT RCT RCT RCT	*** ***** *** *** ****
7	5	USA	*N*_t_ 22	15.4 (1.8)	None	QUAND	****
8	8 20 34 70	USA	*N*_t_ 70 *N*_c_ 66	_t_ 8.5 (1.8) _c_ 8.9 (1.9)	Active	RCT RCT RCT^a^ RCT^a^	* ** *** *****
9	132	USA	*N*_t_ 20 *N*_c_ 20	_t_ 9.2 (1.9) _c_ 8.7 (1.8)	TAU	RCT^a^	***
10	10	UK	*N*_t_ 20 *N*_c_ 15 N-qual: 14 parents 17 children 16 facilitators 5professionals	_t_ 10.2 (2.1) _c_ 10.9 (2.7)	TAU	MIXED (fRCT and QUAL)	***
11	11 19 31 48	USA	*N*_t_ 123 *N*_c_ 66	11.54 (–)	Active	RCT RCT RCT RCT	* *** – **
12	21	USA	*N*_t_ 49 *N*_c_ 19	Not stated	Active	RCT^a^	*
13	201 16	GER	*N*_t_ 50 *N*_c_ 50 Nqual: 18 parents 22 young people	_t_ 11.7 (2.8) _c_ 12.0 (2.9)	TAU	RCT^a^ QUAL	*** *****
14	18 29 72 82 105 116 122 138	USA	*N*_t_ 90 *N*_c_ 90	11.5 (2.0)	Active	RCT^a^ RCT RCT RCT RCT RCT RCT RCT	* ** ***** *** *** ** ** ****
15	26	GER	*N*_t_ 41 *N*_c_ 26	_t_ 10.1 (2.3) _c_ 10.3 (2.7)	TAU	NRCT^a^	****
16	45	Canada	*N*_t_ 19	9.7 (2.6)	None	QUAND	*****
17	62	USA	*N*_t_ 16 *N*_c_ 8	_t_ 14.7 (1.8) _c_ 14.0 (1.7)	Active	fRCT	*
18	67	AUS	*N*_t_ 89	10.4 (2.4)	None	QUAND	****
19	71	UK	*N*_t_ 69 *N*_t_ 71 *N*_c_ 71	6–13 years	Active	RCT^a^	****
21	91	USA	*N*_t_ 13	9–18 years	None	MM (QUAND and QUAL)	**
22	101	USA	*N*_t_ 16 *N*_c_ 14	12–15 years	TAU	RCT^a^	**
23	109	USA	*N*_t_ 10	9–16 years	None	QUAND	****
24	118	GER	375^b^	5.3 ( −)	TAU	NRCT	**
25	135	USA	*N*_t_ 9	11.0 (1.9)	None	MM (QUAND and QUAL)	*****
26	143	Spain	*N*_t_ 15 *N*_c_ 16	10.6 ( −)	Passive	NRCT	***
27	146	USA	*N*_t_ 98 *N*_c_ 69	11.2 ( −)	TAU	RCT^a^	**
28	173 176	USA	*N*_t_ 77 *N*_c_ 58 parents	10.4 (2.4)	TAU	RCT RCT	** ***
29	164	UK	*N*_t_ 20	7–14 years	None	QUAND	–
30	200	USA	*N*_t_ 45 *N*_c_ 49	_t_ 14.4 (1.4) _c_ 14.7 (1.5)	TAU	RCT^a^	***
31	92	USA	*N*_t_ 25 *N*_c_ 18	_t_ 12.6 ( −) _c_ 10.8 ( −)	TAU	fRCT	*****
32	208	USA	*N*_t_ 43 *N*_c_ 44	_t_ 10.4 (3.3) _c_ 10.9 (3.3)	TAU	RCT	***
33	6 202	USA	*N*_t_ 61 youth *N*_c_ 66 youth	13.2 (2.7) 13.3 (2.5) 9–17 years	Active	RCT RCT	***** ****
34	205 211	China	*N*_t_ 37 *N*_c_ 74	8–18 years	Active	NRCT NRCT	** ***
35	212	Iran	*N*_t_ 30 *N*_c_ 30	8–16 years	TAU	NRCT	***
36	94	USA	NC_t_ 21 NC_c_ 19	*M* = 12.2 (2.8) range 9–17	Active	RCT	*****
37	2	Sweden	Nqual: 8 parents 7 children	8–17 years	None	QUAL	*****
38	15	AUS	15 parents 8 children 6 siblings 6 clinicians	*M* = 13.1 (range 9–17)	na	QUAL	*****
40	59 214	UK	5 parents 6 children 7 service users 10 facilitators 101 parent feedback form 138 young people feedback form	4–16 years	na	QUAL MM	**** ****
41	79	UK	36 parents 37 children 30 facilitators	8–17 years	na	QUAL	*****
43	206	IRE	23 parents 7 partners 15 children	5–18 years	na	QUAL	*****
44	213	AUS	10 parents	M(SD) = 8.5 (1.4)	na	QUAL	*****

Note: sample sizes refer to the number of families included unless otherwise specified. *Nt* sample size treatment group, *Nc* sample size control group, *TAU* treatment as usual, *RCT* randomised-controlled trial, *fRCT* feasibility or pilot RCT, *NRCT* non-randomised trial, *QUAND* quantitative descriptive study, *QUAL* qualitative study, *MM* mixed method study, *UK* United Kingdom, *IRE* Ireland, *AUS* Australia, *GER* Germany, *USA* United States of America

^a^Trial included in meta-analysis

^b^Sample size not split by group

### Types of whole-family interventions and components

Most interventions (46%; 14/30) addressed parental depression or substance misuse (30%; 9/30). The remaining interventions addressed families affected by anxiety disorders, bipolar disorders, or multiple disorders. Children needed a diagnosis or symptoms of mental illness to be included in seven of the interventions. The majority of the interventions (86%; 26/30) were outpatient or community-based, one was inpatient, and three used a combination of settings. Most interventions were manualised (90%; 27/30) and had a duration of 3 weeks up to 6 months (80%; 24/30). Two interventions were less than 1 week long, two were up to 9 months, and two were unspecified or open-ended. Fifteen (50%; 15/30) of the interventions worked with families individually, thirteen (43%; 13/30) interventions were delivered to groups of families, and two (6%; 2/30) interventions included both individual and group components.

We regarded interventions as having a family component when they included sessions or activities where members from at least two different levels (e.g., parent vs child level) in the family system were involved. Three interventions (10%; 3/30) had no family component where parents and children received treatment separately. Eight interventions (26%; 8/30) consisted of only family components, where all sessions involved a minimum of parents/caregivers and their children. Other interventions offered a combination of parent or child-only components with whole-family components. Intervention programmes differed in terms of who they involved as part of the family, three interventions (10%; 3/30) included parent–child dyads (mostly mothers), and another three (10%; 3/30) involved parent(s) and one child. The remaining interventions (70%; 21/30) stated that siblings, partners, and other family members were invited to take part. Table 2 gives a summary of the intervention characteristics and Table 3 provides an overview of the family components per study and which family members had been involved.

**Table 2:** Summary of all included interventions

Intervention	Number of papers included (Report ID)	Study ID	Evaluation method	Country	PMI	Aim	Child diagnosis/symptoms	Who delivers intervention	Setting of intervention	Group or individual	Length of intervention	Manualised	Intervention components		
													Parent-only	Child-only	Whole-family
Adolescent coping with Stress Course—adapted	1 (200)	30	Quantitative	USA	Depression	Adolescents taught cognitive restructuring techniques, to identify and challenge irrational unrealistic or overly negative thoughts, with a special focus on beliefs related to having a depressed parent	Yes	Therapists (Masters level)	Outpatients	Group	15 sessions	Yes	3 sessions	15 sessions	–
CBT with mother–child interaction (CBT-MCI)	1 (71)	19	Quantitative	UK	Anxiety disorders	Designed to target potentially anxiogenic maternal parenting behaviours. Specifically, it aims to enhance maternal autonomy promoting cognitions and behaviours and reduce potentially anxiogenic behaviours	Yes	Qualified clinical psychologists or cognitive–behaviour therapists	Outpatients	Individual	8 weeks	Yes	8 sessions	8 sessions	2 sessions
Enhanced CBT intervention	1 (213)	44	Qualitative	Australia	Anxiety disorders	Aims to make systemically informed enhancements to address identified bidirectional maintenance factors of anxiety-related parenting behaviours and cognitions, delivered alongside concurrent treatment of child anxiety disorder via a parallel child-focussed manual	Yes	Clinical psychologist	Community	Individual	10 weeks	YES	10 sessions	10 sessions	–
Coping and Promoting Strength (CAPS)	5 (8, 20, 34, 70, 132)	8, 9	Quantitative	USA	Anxiety disorders	Targets theory-based modifiable child and parent risk factors such as child social avoidance/withdrawal, maladaptive cognitions, and deficits in problem-solving, skills, and anxiety-enhancing parenting behaviours	No	Trained therapists	Community	Individual	6–8 weeks, plus 3 monthly booster sessions	Yes	2 sessions	–	6 sessions
Ecologically based family therapy (EBFT)	5 (11, 19, 31, 48, 21)	11, 12	Quantitative	USA	Substance misuse	Family systems therapy that targets specific dysfunctional interactions linked to the development of problem behaviours	No	Trained therapists	Home-based, office-based	Individual	not specified	Yes	–	–	12 sessions
Entwicklungsförderung in Familien: Eltern- und Kinder-Training in emotional belasteten Familien (EFFEKT-E)	1 (118)	24	Quantitative	Germany	Depression	Trains parenting behaviour and children’s social competence to prevent general child behavioural problems	No	Professionals	In-patient	Individual	3 weeks	Yes	6 sessions	5 sessions	1 session
Family Competence Programme (FCP), an adaptation of the Strengthening Families Programme	1 (143)	26	Quantitative	Spain	Substance misuse	Multi-component programme that aims to reduce the influence of risk factors associated with alcohol and drug use while increasing children’s resilience by reinforcing the main protective factors	No	Trained therapists	Outpatients	Individual	14 weeks	Yes	14 sessions parallel	14 sessions parallel	After each session
Family-focused treatment	3 (6, 94, 202)	33, 36	Quantitative	USA	Bipolar disorder	The goals are to assist youth and family members to recognise and intervene early with symptoms of mood episodes and enhance intrafamilial communication and problem-solving	Yes	Trained professionals	Community	Individual	12 sessions, 4 months	Yes	–	–	12 sessions
Family Friendly Programme (FFP)	1 (212)	35	Quantitative	Iran	Anxiety Disorders	Aims to reduce anxiety symptoms in parents and children. The goal of this program is to create confident parents who know how to build confidence in their child and how to calm the child in times of turmoil and fear	Yes	Trained professionals	Outpatients	Individual	11 weeks	Yes	–	–	11 sessions
Family Group Cognitive-Behavioural	10 (16, 18, 29, 72, 82, 138, 116, 105, 122, 201)	13, 14	Both	USA, Germany	Depression	Aims to prevent MDD and internalizing and externalising symptoms in high-risk youth	No	Clinical social workers and Clinical psychology graduate students	Community	Group	6 months	Yes	12 sessions parallel	12 sessions parallel	As part of each session, plus 2 booster sessions
Family Talk Intervention	13 (1, 2, 93, 119, 123, 148, 162, 177, 178, 179, 180, 186, 206)	1, 3, 4, 5, 6, 37, 43	Both	Sweden, USA, Greece, Germany, Finland, Ireland	Psychosis, affective disorders, depression	Aims to provide information about the parent’s mental illness, reduce the child’s feelings of guilt, and support the child’s relationships within and outside the family	No	Trained professionals	Outpatients	Individual	6 sessions, over 6–9 months	Yes	4 sessions	1 session	1 session
Family Talk Intervention (Group Version)	1 (73)	2	Quantitative	Germany	Depression	Aims to help family members make sense of parental depression through improved communication and deeper understanding of mental illness and its impact on family members	No	Trained mental health professionals	Community	Group	3 months	Yes	2 sessions	5 sessions	1 session for each family
Focus on Families	2 (173, 176)	28	Quantitative	USA	Substance misuse	The programme addressed risk factors for relapse among opiate addicts and risk and protective factors for drug abuse among their children	No	Trained Social workers	Community	Group	9 months	No	20 sessions	–	12 sessions
Fortalezas Familiares (Family Strengths), adapted from KFS	1 (91)	21	Quantitative	USA	Depression	Aims to enhance the resources families must cope with maternal depression by improving communication and families’ understanding of depression and negative family interactions, building parenting competence and confidence, and promoting children’s positive coping strategies and efficacy	No	Professional psychologist and a community mental health professional	Community	Group	12 weeks, plus 2 booster sessions	Yes	–	–	12 sessions, plus 2 booster sessions
Kanu Intervention	1 (26)	15	Quantitative	Germany	Depression	Designed to address several psychosocial challenges related to parental mental illness, such as impaired parent–child interactions, maladaptive social and communication skills, adverse parenting behaviour, and low social support	No	Trained clinicians from both child and adult services (i.e., psychiatrists, psychologists and social workers)	Mixed	Group and individual components	6 months, 10 sessions	Yes	10 sessions	10 sessions	10 sessions
Keeping Families Stronger Intervention (KFS)	1 (109)	23	Quantitative	USA	Depression	Targets the family’s understanding about depression, communication patterns, parenting skills and confidence, positive family experiences and family cohesion, as well as children’s coping skills	No	Mental health clinicians	Outpatients	Group	4 months	Yes	10 sessions	10 sessions	Activity prior to each session, plus 2 booster sessions
KidsTime	2 (59, 214)	40	Qualitative	UK	Multiple	Overall purpose is to reduce the likelihood of children of parents with mental illnesses developing emotional difficulties later on in life	No	Mental health and social care professionals	Community	Group	Open-ended	Yes	Open-ended	Open-ended	Open-ended
Mobile-enhanced family-focused therapy (FFT)	1 (5)	7	Quantitative	USA	Bipolar disorder	Use a mobile app to encourage learning and implementation of family-focused therapy skills as well as facilitate information exchange between clinicians, teens and families in family-focused therapy using an interactive mobile app	Yes	Trained clinicians	Community and Online (App)	Individual	4 months	Yes	–	–	12 sessions
Moving Parents and Children Together Programme (M-PACT)	1 (79)	41	Qualitative	UK	Substance misuse	The content is focused on improving relationships between parents and children, such as exploring communication, parenting and asking families to develop a toolbox of strategies and activities to draw upon (as individuals and as families) in difficult times	No	Trained facilitators	Community	Group	8 weeks	Yes	–	–	8 sessions, plus 2 reunion sessions
Multi Family Therapy	2 (205, 211)	34	Quantitative	China	Depression	The intervention targeted theory-driven modifiable child and parent anxiety risk factors through the acquisition of CBT skills. Children are taught to reduce anxiety by practising relaxation strategies, behavioural exposure, cognitive restructuring, and problem-solving. Parents were taught to reduce anxiety-promoting parenting behaviours	No	Mental health social workers	Community	Group	3 months, 42 contact hours	No	–	–	4-day programme, plus 2 half day reunions
Multisystemic Therapy-Building Stronger Families Programme (MST-BFP)	2 (92, 208)	31, 32	Quantitative	USA	Substance misuse	An integrated treatment model designed to comprehensively address co-occurring parental substance abuse and child maltreatment among families involved in the child welfare system and to overcome barriers to service access and treatment coordination for this population	No	Therapists	Community	Group and individual components	6–9 months	Yes	Individualised activity	Individualised activity	Individualised activity
Parent-Adolescent CBT (PA-CBT) protocol	1 (62)	17	Quantitative	USA	Depression	Aims to teach both the parent and the adolescent problem-solving in cognitive behavioural therapy (CBT)	Yes	Therapists (Masters and PhD Level)	Outpatients	Individual	24 weeks	Yes	18 sessions in parallel	18 sessions in parallel	Number unspecified
Prevention Intervention Programme (PIP), adapted from Family Talk Intervention	1 (135)	25	Both	USA	Depression	Designed to enhance strength and resilience in children and young people whose parents have depression	No	Professionals	Outpatients	Individual	3–7 months	Yes	4 sessions	1 session	1 session
Project Hope	1 (101)	22	Quantitative	USA	Depression, substance misuse	The goal is to prevent adolescent depression and substance use, as well as their co-occurrence, by strengthening parenting and family relationships and enhancing youth resilience	No	Professionals	Outpatients	Individual	10 sessions	Yes	3 sessions	1 session	5 sessions
Strength to Strength, adapted from Family Talk Intervention	1 (164)	29	Quantitative	UK	Depression	Seeks to maximise the resilience of children at high risk of developing subsequent mental health difficulties	No	Mental health professionals	Community	Group	6 months	Yes	Not specified	Not specified	Not specified
Strong African American Families (SAAF) programme	1 (146)	27	Quantitative	USA	Depression	Programme targets regulated-communicative parenting including consistent discipline, parental monitoring, and open communication. The programme targets youth intrapersonal factors including academic competence, social competence and self-regulation	No	Trained facilitators	Community	Individual	7 weeks	No	7 sessions in parallel	7 sessions in parallel	Within each of the 7 sessions
Supporting Kids and Their Environment (SKATE)	1 (67)	18	Quantitative	Australia	Substance misuse	The overall aim of the intervention was to reduce behavioural problems and improve family functioning by promoting optimal development of children who have a substance dependent parent	No	Trained group facilitators	Community	Group	8 weeks	Yes	–	8 sessions	Homework task each week
The Family Model	1 (15)	38	Qualitative	Australia	Multiple	The aim of the conversation between clinician and family members is to develop a shared understanding about the impact of symptoms and associated responses	No	Trained Psychiatrist	Outpatients	Individual	One-off	Yes	–	–	1 session
The Renascent Children’s Programme	1 (45)	16	Quantitative	Canada	Substance misuse	The overaching goals are to create a safe environment for children to learn about addiction and how it impacts their family, help foster coping skills, and increase emotional and psychological well-being through peer-support	No	Trained professionals	Community	Group	4 days	Yes	Activities over 4 days in parallel	Activities over 4 days in parallel	Crossover activities over 4 days
Young Smiles	1 (10)	10	Both	UK	Multiple	Aims to improve the health-related quality of life of children and adolescents living with serious parental mental illness	No	Trained Practitioners	Community	Group	8 weeks	Yes	5 sessions	8 sessions	–

*PMI* parent mental illness

**Table 3:** Overview of family components

Study ID	Report ID	Country	Intervention	*N* families	Family participants (treatment)	Family participants (control)	Family component
1	1	Greece	Family Talk Intervention	*N*_t_ 30 *N*_c_ 32	Fathers 6 Mothers 26 Male offspring 14 Female offspring 16	Fathers 6 Mothers 24 Male offspring 19 Female offspring 13	Parent + partner + child take part One parents + child session per child. No sessions with siblings or other family members
2	73	GER	Family Talk Intervention (Group Version)	*N*_t_ 28 *N*_c_ 9	Father – Mother – Male offspring 12 Female offspring 16	Father – Mother – Male offspring 4 Female offspring 5	One individual family session with parents and child. No information on involvement of siblings or other family members
3	93 119 123	Finland	Family Talk Intervention	*N*_t_ 53 *N*_c_ 56	Father – Mother – Offspring 76	Father – Mother – Offspring 69	Parent + partner + child One individual family session with parents and child. No information on involvement of siblings or other family members
4	186	USA	Family Talk Intervention	*N*_t_ 7	Parent 7 Partner 7 Offspring 8	na	1–2 Family sessions. No information on involvement of siblings or other family members
5	180	USA	Family Talk Intervention	*N*_t+c_ 28	Parents 54 Average number of children in family 2	na	1–2 Family sessions. No information on involvement of siblings or other family members
6	148 162 177 178 179	USA	Family Talk Intervention	*N*_t_ 55 *N*_c_ 44	Parents 97 Offspring 78	Parents 76 Offspring 54	1–2 Family sessions. No information on involvement of siblings or other family members
7	5	USA	Mobile-enhanced family-focused therapy (FFT)	*N*_t_ 22	Mother 16 Father 17 Male offspring 10 Female offspring 12	na	12 Family sessions with child and parent(s). Other family members are invited to take part
8	8 20 34 70	USA	Coping and promoting strength	*N*_t_ 70 *N*_c_ 66	Mothers 107 across both groups Fathers 29 across both groups 113 offspring across both groups		6 Sessions for all interested family members
9	132	USA	Coping and promoting strength	*N*_t_ 20 *N*_c_ 20	Parent 20 Partner – Offspring 20	Parent 20 Partner – Offspring 20	6 Sessions for all interested family members
10	10	UK	Young SMILES	*N*_t_ 18 *N*_c_ 15	Parent 18 Partner – Male offspring 12 Female offspring 12	Parent 15 Partner – Male offspring 7 Female offspring 9	None
11	11 19 31 48	USA	Ecologically based family therapy (EBFT)	*N*_t_ 123 *N*_c_ 60	Mothers 123 across both groups Offspring 180 across both groups 36% included siblings across both groups 9% included partner across both groups 10% other family member across both groups		All children, partners and other caregivers invited. 12 family sessions focus on needs assessment, problem-solving, communication
12	21	USA	Ecologically based family therapy (EBFT)	*N*_t_ 49 *N*_c_ 19	Mothers 49 Partners – Offspring –	Mothers 19 Partners – Offspring –	All children, partners and other caregivers invited. 12 family sessions focus on needs assessment, problem-solving, communication
13	201 16	GER	Family Group Cognitive-Behavioural	*N*_t_ 50 *N*_c_ 50	Parent 50 Offspring 50 Siblings 77%	Parent 50 Offspring 50 Siblings 72%	The first two sessions involve parents and children for the whole session, the remaining 6 sessions start and end in the family- and group- setting but include separate activities for parents and children. Siblings are allowed to join
14	18 29 72 82 105 116 122 138	USA	Family Group Cognitive-Behavioural	*N*_t_ 90 *N*_c_ 90	Mothers 160 across both groups Fathers 20 across both groups Female offspring 121 across both groups Male offspring 121 across both groups		8–12 Sessions where parents and children meet together part of the time and separately part of the time. No information on involvement of siblings or other family members
15	26	GER	Kanu Intervention	*N*_t_ 41 *N*_c_ 26	Parents 46 Partner – Offspring 60	Parents 25 Partner – Offspring 44	10 Individual sessions involving parent (and partner) and children
16	45	Canada	The Renascent Children’s Programme	*N*_t_ 19	Mother 15 Father 6 Grandmother 3 Uncle 1 Stepparent 1 Male offspring 12 Female offspring 14	na	Parent, guardian or other family member can attend. Generally separate groups for parents and children with overlapping activities over 4 days. No specific information about the activities available
17	62	USA	Parent-Adolescent CBT (PA-CBT) protocol	*N*_t_ 16 *N*_c_ 8	Parent 16 Offspring 16	Parent 8 Offspring 8	All 18 individual sessions with child concluded with a conjoint “check-in” meeting between parent and teen to enhance positive communication
18	67	AUS	Supporting Kids and Their Environment (SKATE)	*N*_t_ 89	Parents – Female offspring 51 Male offspring 38	na	Letters sent to parents with tasks that children and parents work on together
19	71	UK	CBT with mother–child interaction	*N*_t_ 71 *N*_c_ 69 *N*_c_ 71	All mothers Offspring 71	All mothers Offspring 71 Offspring 69	Two sessions with mother and child completing task that are video recorded for later video-feedback
21	91	USA	Fortalezas Familiares (family strengths)	*N*_t_ 13	Mothers 13 Fathers 10 Grandmother 1	na	Other caregivers or children are invited to take part. All 12 sessions include all family members
22	101	USA	Project Hope	*N*_t_ 16 *N*_c_ 14	Parent – Partner – Offspring –	Parent – Partner – Offspring –	3 Sessions for parents and target child and 3 sessions for the whole family
23	109	USA	Keeping Families Stronger Intervention	*N*_t_ 10	Mothers 10 Partner 3 Children 16	na	Partners and children were invited to take part. Families attended 10 weekly multi-family sessions involving separate but concurrent parent and child groups and one individual family session. Home projects for families
24	118	GER	Eltern- und Kinder-Training in emotional belasteten Familien EFFEKT-E	375^a^	Mothers had average of 1.07- 1.99 children Partners –	Not reported	Separate child and parent sessions and 1 mother–child session, where child and mother practise content learned together. No information on involvement of partners or other family members
25	135	USA	Prevention Intervention Programme (PIP)	*N*_t_ 9	Mothers 9 Partners 5 Male offspring 4 Female offspring 5 Siblings –	na	One family session with main aim to increase shared understanding. Who takes part in family session is defined by families themselves
26	143	Spain	Family Competence Programme	*N*_t_ 15 *N*_c_ 16	Parents 28 Offspring 19	Parents 30 Offspring 16	Over 14 sessions, families have lunch together, then separate parent and child groups (1 h). In second half (1 h), parents and children practise together. “Entire family” is invited to take part
27	146	USA	Strong African American Families (SAAF)	*N*_t_ 98 *N*_c_ 69	Caregiver 98 Average children 2.75	Caregiver 69 Average children 2.84	Seven separate child and parent sessions followed by joint session to practise skills. No information on involvement of partners and other family members
28	173 176	USA	Focus on Families	*N*_t_ 75 *N*_c_ 55 parents	Parents 78 Offspring 97	Parents 57 Offspring 81	Children join 12 family sessions to help families practise new skills. Parents have additional 20 separate sessions. No information on involvement of other family members
29	164	UK	Strength to Strength	*N*_t_ 10	Not reported	na	Family sessions with parents and children form part of intervention. No information about frequency or length of sessions, or involvement of other family members
30	200	USA	Adolescent coping with Stress course	*N*_t_ 45 *N*_c_ 49	Offspring 45	Offspring 49	No family component
31	92	USA	Multisystemic Therapy-Building Stronger Families Programme	*N*_t_ 25 *N*_c_ 18	Mothers 25 Offspring 25 Fathers were excluded from study	Mothers 18 Offspring 18 Fathers were excluded from study	Family is assessed together and defines treatment goals together. Other members in and around family are involved to develop safety plan for child. Individual sessions are delivered based on family members’ needs, thus involvement of family members and activities can vary
32	208	USA	Multisystemic Therapy-Building Stronger Families Programme	*N*_t_ 43 *N*_c_ 44	Parents 51 Mothers 94% Offspring 43 female offspring 47.1%	Parents 46 Mothers 83% Offspring 43 female offspring 48%	Family is assessed together and defines treatment goals together. Other members in and around family are involved to develop safety plan for child. Individual sessions are delivered based on family members’ needs, and thus, involvement of family members and activities can vary
33	6 202	USA	Family-focused treatment	*N*_t_ 61 *N*_c_ 66	Female offspring 37 Male offspring 24	Female offspring 45 Male offspring 21	12 Sessions with child, parent, partner and if possible siblings
34	205 211	China	Multi Family Therapy	*N*_t_ 34 *N*_c_ 27	Mothers 30 Fathers 4 Partner 12 Offspring 45	Mothers 22 Fathers 5 Partner 7 Offspring 18	4-Day programme plus 2 half day reunions that include multiple families and their members
35	212	Iran	Family Friendly Programme	*N*_t_ 30 *N*_c_ 30	Parents 30 Offspring 30	Parents 30 Offspring 30	11 Sessions to enhance parent and child relationship using elements from parent–child interaction therapy and mindfulness-based cognitive therapy. No information about involvement of partners, siblings, or other family members
36	94	USA	Family-focused treatment	NC_t_ 21 NC_c_ 19	Parents – Female offspring 10 Male offspring 11	Parents— Female offspring 7 Male offspring 12	Involved parent, partners, children, and siblings in 12 sessions
37	2	Sweden	Family Talk Intervention	Not reported	8 parents 7 children	na	1–2 Family sessions. No information on involvement of siblings or other family members
38	15	AUS	The Family Model	Not reported	15 parents 8 children 6 siblings	na	1 Session with family and clinician about impact of illness
40	59 214	UK	Kidstime	Not reported	5 parents 6 children	na	Family sessions with separate parent and child sessions and shared dinner in the end
41	79	UK	Moving Parents and Children Together Programme	Not reported	36 parents 37 children	na	Eight sessions that involve multiple families, some sessions include separate activities for children and parents and some for the family unit
43	206	IRE	Family Talk Intervention	Not reported	23 parents 7 partners 15 children	na	1–2 Family sessions. Clinician meets with whole family including siblings. No information on involvement of other family members
44	213	AUS	Enhanced CBT Intervention	Not reported	10 parents	na	Parents and children get parallel treatment and learn about bidirectional impact of anxiety in parent and child. No joint sessions

Note: *N*_t_ sample size treatment group, *N*_c_ sample size control group, *na* not applicable

Intervention components were grouped into five higher level component characteristics: (1) structural components, (2) components from psychotherapeutic frameworks, (3) skills training, (4) psychoeducation, and (5) building resources. Twenty-four of the 30 interventions (80%; 24/30) included one or more of the identified structural components, although it varied what structural components programmes included.

All interventions delivered regular sessions, with two interventions being open-ended and the rest following a fixed schedule. Approximately one-third of the interventions (30%; 10/30) set homework tasks or encouraged family members to practise between sessions. Some interventions (30%; 9/30) facilitated parent–child interactions by stimulating parents to spend quality time with their child or by creating positive parent–child moments in the sessions. Relatively few interventions included an assessment (40%; 12/30) or goal setting component (20%;6/30). Most interventions (76%; 23/30) drew on one or more psychotherapeutic frameworks, such as cognitive behaviour therapy or systemic family therapy. The most common frameworks were multi-family or group therapy (46%; 14/30), and cognitive behavioural therapy (50%; 15/30). Many also contained elements of play or creativity (30%; 9/30), such as hand puppets (ID-*n*:118) and drawing (ID-*n*:67).

All but one intervention, namely Multiple Family Therapy (ID-*n*: 205, 211), contained one or more skills training components. The majority of interventions taught problem-solving and coping skills (76%; 23/30) such as relaxation and breathing exercise; communication skills (67%; 20/30), and/or parenting skills (60%; 18/30). Some interventions (30%; 9/30) specifically focused on supporting families with talking about parental mental illness in the family.

Most interventions (90%; 27/30) included one or more psychoeducational components, with the majority (70%; 21/30) providing psychoeducation on mental illness. Almost half of the interventions (50%; 15/30) provided psychoeducation on the impact of parental mental illnesses on children and other family members.

Increasing support and resources for families, such as developing a family care plan or strengthening the family’s network, was provided by fewer than half of the interventions (46%; 14/30). Some interventions aimed to build support networks for children (36%; 11/30) and parents (26%; 8/30) by identifying sources of support (ID-*n*:10) and encouraging positive friendships (ID-*n*:143). Interventions also linked and signposted participants with other potentially helpful services (40%; 12/30), such as social services (ID-*n*:123). A detailed overview of the intervention components can be found in the Supplement materials 2.

### Effectiveness of whole-family interventions

Thirty-six independent studies provided quantitative data on child, parent, and family outcomes. Table 4 provides an overview of the results for the included studies per outcome category. Of the 36 studies, 28 studies (77%; 28/36) assessed changes in child mental health outcomes, 15 (41%; 15/36) in parental mental health outcomes, and 27 (75%; 27/36) in family outcomes. We focussed on the following child, parent, and family outcomes: child internalising problems (i.e., anxiety, depression, and suicidality), child externalising problems (i.e., behavioural problems, conduct problems, and attention-deficit hyperactivity disorder), parent symptoms of mood and anxiety disorders (here summarised as internalising), parental substance abuse, other parental psychological symptoms (i.e., psychological distress and global functioning), family functioning (i.e., spouse relationship, sibling relationship, family communication, family conflict, family times, and routines), parenting (i.e., parenting stress, parenting sense of competence, parenting skills, parenting style, and child abuse), and assessments of the parent–child relationship (i.e., communication, and parent–child conflict or observation of interactions).

**Table 4:** Overview of results for intervention outcomes per study

Study ID	Report ID	Improved child outcomes			Improved parent outcomes				Improved family outcomes			
		Measure	INT	EXT	Measure	INT	Drug abuse	Other	Measure	Family functioning	Parenting	Parent–child relationships
1	1	SDQ, CDI, SCARED	+	/	BDI-SF, SSAI	+	/	/	FAD-GF	+ +	/	/
2	73	CBCL, SDQ	0	+ +	/	/	/	/	/	/	/	/
3	93 119 123	CDI, BDI, SDQ	+	0	/	/	/	/	Interview	+ + /0	+ /0	/
4	186	/	/	/	/	/	/	/	DAS, Interview	0	/	/
5	180	/	/	/	/	/	/	/	interview	+ + /0	/	/
6	148 162 177 178 179	YSR, CDI, CBCL	+	/	/	/	/	/	Interview	+ + /0	/	+ +
7	5	CDRS-R PHQ-9	+	/	/	/	/	/	/	/	/	/
8	8 20 34 70	CBCL	+ + /0	/	/	/	/	/	PSI-SF, CPIC, other	0	0	/
9	132	ADIS SCARED	+ + /0	/	/	/	/	/	/	/	/	/
10	10	/	/	/	/	/	/	/	A–O’Leary parenting scale, PSI-SF	/	+ ^a^	/
11	11 19 31 48	/	/	/	Form-90	/	+ + / +	/	PBI, ARCS	/	+ +	+ /0
12	21	/	/	/	Form-90, BDI-II	+ +	+ +	/	ARCS	/	/	+ + /0
13	201	YSR, CBCL, DIKJ	+ /0	0	BDI-II	+	/	/	ESI	/	0	/
14	18 29 72 82 105 116 122 138	CBCL, YRS, CES-D	+ + /0	+ + /0	BDI-II, SCID	+ + /0	/	/	IFIRS	/	+ +	/
15	26	SDQ, CDI	+ +	/	/	/	/	/	PI-C	/	/	+ + /0/–
16	45	SDQ, SMFQ	+	+	/	/	/	/	PSQ, FACES-IV	+ /0	+ /0	/
17	62	BSS, BDI	+ /0	/	BDI-II, SCID-I/P	+ + /0	/	/	/	/	/	/
18	67	CBCL	+	+	/	/	/	/	FSS	+	/	/
19	71	SCAS, CAIS, SMFQ, SDQ	+ /0	0	/	/	/	/	/	/	/	/
21	91	SDQ	+	+	BSI-18, FAD	+	/	+	APQ, FTRI, CRPBI, CBQ	+	+	/
22	101	MFQ	0	/	/	/	/	/	APQ, Interview, other	0/–	+ + /0	0
23	109	BASC	+	+	BSI-18	+	/	+	MFAD, FTRI; DAS; CRPBI	+	+	/
24	118	VBV	+	+ /0	/	/	/	/	APQ;PSO; PSI	/	+ + /0	/
25	135	CBCL	0	0	GAS	/	/	+	/	/	/	/
26	143	BASC	+	+	/	/	/	/	SFP	+ + / + /0	+ /0	/
27	146	/	/	/	CES-D	+ +	/	/	self-developed scale	/	+ +	/
28	173 176	Amount drug use	/	+	drug use self-report	/	+ + /0	/	MFCS	+ + /0	/	/
29	164	SDQ	+	0	/	/	/	/	FACES, other	+ /0	/	0
30	200	CES-D, HAM-D, CBCL	+ + /0	0	/	/	/	/	/	/	/	/
31	92	TSCC	+ /0	0	ASI-V, BDI-II	+	+	/	CTS	/	+ + / + /0	/
32	208	/	/	/	ASI-V	/	+ + / +	/	CTS-PC; APQ	/	+ /0	/
33	6 202	SIQ, A-LIFE	+ /0	/	/	/	/	/	CBQ	/	/	+ +
34	205 211	OYPFS	0	0	BSI-18	+	/	+	PCR; PSOC; PSI; GFFS	+ /0	+ /0	0
35	212	SCARED	+ +	/	ASI-R	+ +	/	/	/	/	/	/
36	94	YMRS, CDRS-R	0	+ ^	/	/	/	/	/	/	/	/
40	214	/	/	/	/	/	/	/	feedback form	+ ^b^	/	/
**Total group effect** (+ +)			2	1		3	1	0		1	3	2
**Total mixed effects** (+ /0)			9	2		2	3	0		9	8	3
**Total time effect only** ( +)			11	7		6	1	4		4	3	0
**Total null effect** (0)			5	8		0	0	0		2	2	3
**Total number effects**			27	18		11	5	4		15	16	8

**Note**: +  + : significantly better outcomes in the intervention group compared to the control group; + : positive significant change in the intervention group, but no difference with the control group, or no control group present; 0: no significant finding on this outcome reported; − : negative significant change in the intervention group; –: significantly better outcomes in the control group compared to the intervention group. A combination of these signs represent varying findings due to multiple outcome measures, multiple reporters, multiple time-points, or reporting of subgroup analyses. ^this study assessed manic symptoms which were grouped into EXT for this table

*INT* internalising symptoms, *EXT* externalising symptoms, **Child outcome measures** = *ADIS* Anxiety Disorder Interview Schedule, *A-LIFE* Adolescent Longitudinal Interval Follow-up Evaluation, *BASC* Behaviour Assessment System for Children, *BDI* Beck’s Depression Inventory, *BSS* Becks Scale for Suicide Ideation, *CAIS* The Child Anxiety Impact Scale, *CBCL* Child Behaviour Checklist, *CES-D* Center for Epidemiologic Studies Depression Scale, *CDI* Child Depression Inventory, *CDRS-R* Children’s Depression Rating Scale-Revised, *DIJK* Depression in Jugend und Kindern, *HAM-D* Hamilton Depression Scale, *MFQ* Mood and Feeling Questionnaire, *OYPFS* Ohio Youth Problems and Functioning Scales, *PHQ-9* Patient and Health Questionnaire, *SCARED* Screen for Child Anxiety Related Disorders, *SCAS* Spence Children’s Anxiety Scale, *SDQ* Strengths and Difficulties Questionnaire, *SIQ* Suicidal Ideation Questionnaire, *SMFQ* Short Mood and Feeling Questionnaire, *TSCC* Trauma Symptom Checklist for Children, *VBV* Verhaltensbeobachtungsbogen für Vorschulkinder, *YMRS* Young Mania Rating Scale, *YSR* Youth Self Report Questionnaire. **Parent outcome measures** = *BDI-SF* Beck Depression Inventory—Short Form, *BDI-II/BDI-SF* Beck Depression Inventory II/Short Form, *ASI(-R)* Addiction Severity Index, *BSI-18* Brief Symptom Inventory, *CES-D* Center for Epidemiologic Studies Depression Scale, *GAS* Global Assessment Scale, *SCID-I/P* Structured Clinical Interview for DSM (I-Research version). **Family outcome measures** = *APQ* Alabama parenting questionnaire, *ARCS* Autonomy and Relatedness coding system, *CBQ* Conflict Behaviour Questionnaire, *CPIC* Children’s Perception of Interparental Conflict, *CRPBI* Revised Child Report of Parenting Behaviour Inventory, *CTS(-PC)* Conflict Tactics Scale—Parent–Child, *DAS* Dyadic Adjustment Scale, *ESI* parenting style inventory, *FACES(-IV)* Family Adaptability and Cohesion Evaluation Scales—Version IV, *FAD-GF* Family Assessment Device—General Functioning subscale, *FSS* Family Support Scale, *FTRI* Family Times and Routines Index, *GFFS* General Family Functioning Scale, *IFIRS* Iowa Family Interaction Rating Scales, *MFAD* McMaster Family Assessment DEvice, *MFCS* Moos Family Cohesion Scale, *PBI* Parenting Behaviour Inventory, *PCR* Parent–Child Relationship scale, *PI-C* Parent–Child relationship Inventory, *PSI(-SF)* Parenting Stress Index—Short Form, *PSOC* Parenting Sense of Competence Scale, *PSQ* Parenting Style Questionnaire, *SFP* Programme Evaluation Battery

^a^This study reported means to decrease over time, but did not include significance testing as it was a feasibility study

^b^This study did not include a baseline assessment but asked participants to rate what changes had occurred due to the intervention (ID-*n* = 186) or whether participants expected the session to help them with specific outcomes (ID-*n* = 214)

#### Child internalising outcomes

Twenty-seven studies (75%; 27/36) assessed changes in children’s internalising symptoms (27%; 10/36), including depression (41%; 15/36) and anxiety symptoms (19%; 7/36). Of these 27 studies, five studies (18%; 5/27) found no intervention effects, nine studies (33%; 9/27) reported mixed findings and 13 studies (48%; 13/27) reported significant post-intervention effects of reduced internalising symptoms in children. Studies reporting no intervention effects were small pilot studies (ID-*t*: 2, 22, 25). Of the 13 studies that indicated any positive intervention effects, 8 studies reported effect sizes of which the majority (*t* = 5) were small-to-medium-effect sizes (*d* = 0.22–0.42). Studies reporting large-effect sizes (*d* = 0.95–1.58) were also either small pilot or feasibility trials (ID-*t*: 7, 16, 26). Seven of the 13 reportedly ‘effective’ studies involved an active control condition of which only two (7%; 2/27) reported significant ‘time × group’ effects (ID-*t*: 15, 35). Three studies that reported significant time effects but no ‘group × time’ effect involved control groups that received a short intervention containing psychoeducational lectures for parents (ID-*t*: 1,3,6). For the other four studies, three control groups received treatment as usual and one received enhanced care containing six additional psychoeducational sessions. Nine studies reported mixed findings (33%; 9/27), where findings differed between reporters (*t* = 5; i.e., parent, clinician, and child), type of self-report measure used (*t* = 4), or effects were temporary or disappeared after controlling for baseline measures. In terms of reporter differences, we found that parents and clinicians tended to report greater changes in children’s internalising levels compared to children themselves.

#### Child externalising symptoms

Fewer studies (50%; 18/36) assessed children’s externalising problems. Eight studies (22%; 8/36) measured changes in behavioural and externalising symptoms, six (16%; 6/36) assessed conduct symptoms, including aggressive behaviour, three studies (8%; 3/36) measured changes in children’s hyperactivity levels, and one study (2%; 1/36) assessed levels of drug use. Of the 18 studies assessing any type of externalising symptoms, eight studies (44%; 8/18) reported some intervention effects, eight studies (44%; 8/18) found no effects and two studies (11%; 2/18) reported mixed findings, where the results differed depending on the scale used (CBCL vs YSR) and the respective reporter (child vs parent). Of the eight studies reporting some form of intervention effect, three studies did not provide descriptive statistics or outcomes of statistical tests (ID-*t* 24, 26, 28). Five studies were small pilot or feasibility trials (ID-*t* 2,16, 21, 23, 26) of which three studies reported small-to-moderate-effect sizes (*d* = 0.39–0.62) and two reported large-effect sizes (*d* = 0.70–0.95); however, sample size in these studies were small.

#### Parental mental health outcomes

Fifteen studies assessed any parental mental health outcomes. For internalising symptoms (*t* = 11), three studies (27%; 3/11) reported significantly better parental internalising outcomes in the intervention group compared to the control group. Six studies (55%; 6/11) reported positive changes over time and two studies (18%; 22/11) reported mixed findings, where findings differed by measurement or time of follow-up assessment. Four of these studies reported effect sizes, which ranged between *d* = 0.71 to *d* = 1.06, thus indicating moderate-to-large intervention effect. However, only one of these studies (ID-*t*:31) had a high-quality rating and included a control group. For parental substance abuse, most studies (80%; 4/5) reported mixed findings and none reported effect sizes. One study (ID-*t*:20) reported positive intervention effects for substance abuse in both the treatment and the TAU group, but the effects differed for certain subgroups, which was linked back to initial referral reasons. Studies assessing other parental mental health outcomes (*t* = 4) reported positive changes; however, they found no significant ‘group x time’ differences (*t* = 1), no control group was present (*t* = 3), and only two studies reported effect sizes (*d* = 0.86 and *d* = 0.93) which albeit large were both small feasibility studies without a control group.

#### Family outcomes

A total of 27 (75%; 27/36) studies assessed family outcomes relating to family functioning (55%; 15/27), parenting behaviour (51%; 14/27), and parent–child relationship (33%; 9/27). For family functioning, five studies (33%; 5/15) reported any positive changes but only one found significant ‘group × time’ effects. Nine (60%; 9/15) studies reported mixed findings, and two (13%; 2/15) reported no effect for family functioning. Six studies (37%; 6/16) reported positive changes in parenting, eight (50%; 8/16) reported mixed findings, and two (12%; 2/16) reported no effects in parenting. For changes in parent–child relationships, two studies (25%; 2/8) reported positive ‘group × time’ changes, three reported mixed findings (37%; 3/8), and three (37%; 3/8) observed no changes in the parent–child relationship. On a few occasions, findings were in favour of TAU, for instance in Project Hope (ID-*t*:22, families receiving TAU indicated better family communication); and in Kanu (ID-*t*:15), the researchers observed that levels of parental rejection were lower in the control group. Only two studies (ID-*t*:21 and 23) reported effect sizes for family outcomes, which suggested moderate-to-large effects for family functioning (*d* = 0.57–1.03) and small-to-moderate effects for parenting behaviours (*d* = 0.03 to *d* = 0.52).

### Meta-analysis

For the meta-analyses, we extracted 63 effect sizes from 12 studies (*t*) with a pooled sample size of *n* = 1298 (*n*_t_ = 681 participants in treatment condition and *n*_c_ = 617 in control conditions). We conducted multiple analyses to distinguish between different levels of child and parent outcomes as well as type of reporter (parent/child) and four different times of follow-up assessment. The below outcomes are reported in ranges to reflect outcomes from the main (*g*_a_) and the sensitivity analysis (*g*_b_).

#### Child mental health outcomes reported by children

**Fig. 1:**
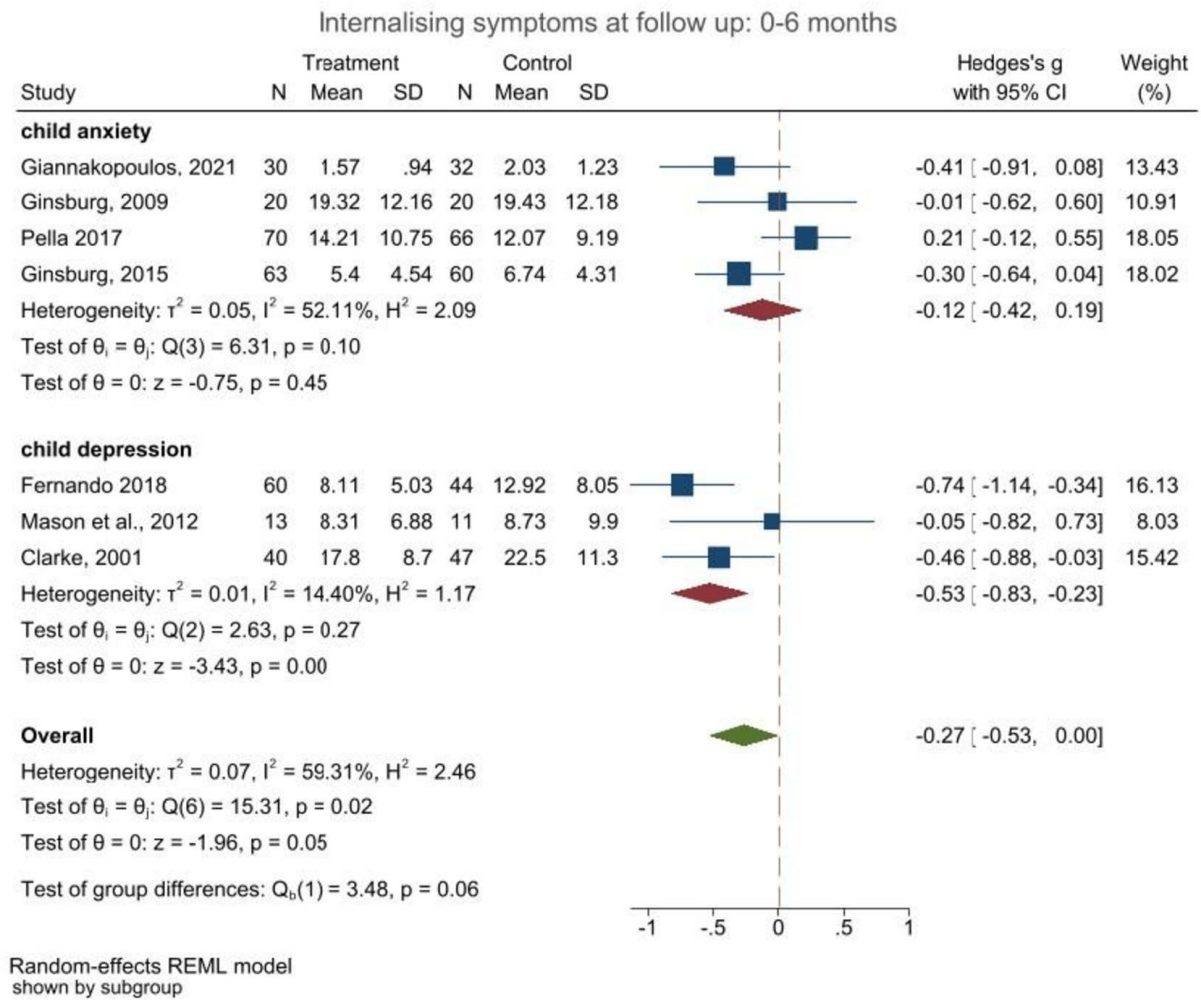
Forest plot child internalising outcomes reported by child at 0–6 month follow-up (a)

Studies with short-term follow-ups (*t* = 4) indicated small intervention effects for child internalising symptoms ranging between *g*_a_ =  − 0.27 (95% CI:  − 0.53,0.00; *p* = 0.050) and *g*_b_ =  − 0.17 (95% CI:  − 0.46, 0.13; *p* = 0.27). Similar trends (*t* = 10) were reported for internalising symptoms assessed between 6 and 10 months post-intervention *g*_a_ =  − 0.18 (95% CI: − 0.55; 0.20, *p* = 0.35) and *g*_b_ =  − 0.20 (95% CI: − 0.55, 0.16; *p* = 0.27). These small intervention trends decreased further in studies (*t* = 4) with longer follow-up times at assessment (10 to 18 months: *g*_a_ =  − 0.05; 95% CI: − 0.27, 0.18; *p* = 0.69 and *g*_b_ =  − 0.02; 95% CI: − 0.28, 0.25; *p* = 0.91; and 18 + months: *g*_a_ =  − 0.03; 95% CI: − 0.22, 0.15; *p* = 0.73 and *g*_b_ =  − 0.04; 95% CI: − 0.15, 0.22; *p* = 0.71). The forest plots (see Fig. 1 and Figs. S2–S8 in supplements) show that only three studies [11–13] consistently reported reduced internalising symptoms, while the remaining studies showed no significant treatment effects. Heterogeneity levels were small-to-medium across all meta-analyses, apart from one, where *I*^2^ = 87.12%–85.51% and *H*^2^ = 7.76–6.90 (see Figs. S1–S8 in supplement materials for all time-points and the sensitivity analyses).

#### Child mental health outcomes reported by parents

**Fig. 2:**
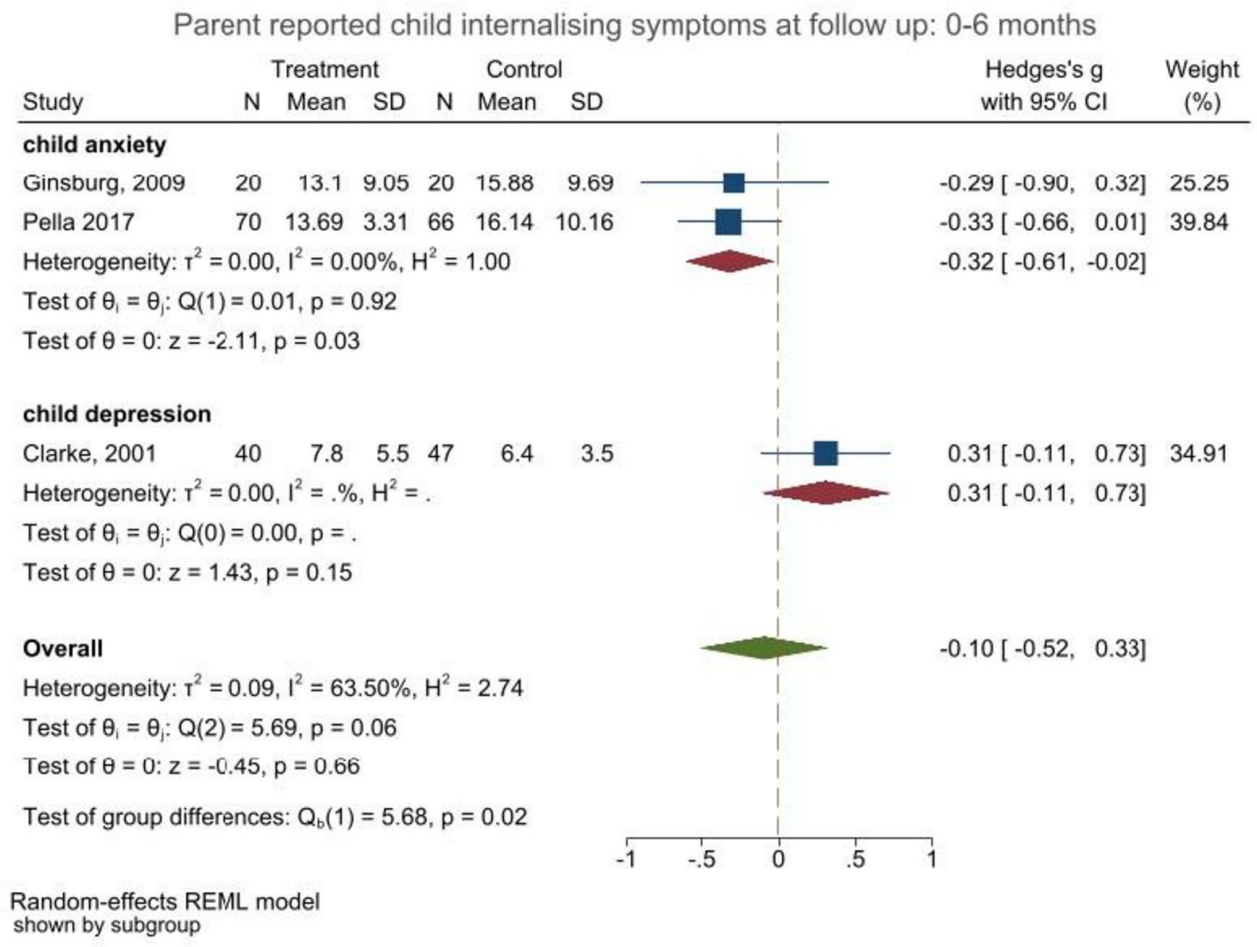
Forest plot child internalising outcomes reported by parents at 0–6 month follow-up (a)

Six studies included data on parent-reported internalising symptoms of children and were included in the following models. Pooled effect sizes ranged between *g*_a_ =  − 0.10 (95% CI: − 0.52, 0.33; *p* = 0.66) and *g*_b_ =  − 0.05; (95% CI: − 0.32, 0.21;* p* = 0.70) for studies with short-term follow-up assessments (see Fig. 2 and Figs. S9 and S10 in the supplements). Meta-analyses of studies reporting medium-term outcomes (*t* = 5) indicated slightly larger, yet small pooled effect sizes ranging between *g*_a_ =  − 0.16 (95% CI: − 0.33, − 0.02; *p* = 0.08) and *g*_b_ =  − 0.18 (95% CI: − 0.36, − 0.01; *p* = 0.04) at 6–10 months follow-up and *g*_a_ =  − 0.22 (95% CI: − 0.65, 0.22; *p* = 0.34) to *g*_b_ =  − 0.17 (95% CI: − 0.50, 0.16; *p* = 0.30) between 10 and 18 month follow-up (Figs. S11–S14). Only three studies reported outcomes for long follow-up assessments (See Figs. S15 and S16), of which one study reported significant findings [14]. Heterogeneity levels were high in all meta-analyses including studies with long-term follow-up assessments (*I*^2^ = 72.32%–89.43% and *H*^2^ = 3.61–9.46). See Figs. S9–S16 in supplement materials for all time-points and sensitivity analyses.

We did not conduct meta-analyses for child externalising symptoms as only three trials reported data for child externalising symptoms, of which only one [17] reported significant findings.

#### Parent mental health outcomes

Six studies reported parental mental health outcomes, of which five reported on medium-term outcomes post-intervention (see Figs. S19 and S20), and the meta-analysis indicated small, non-significant effect sizes ranging between *g*_a_ =  − 0.14 (95% CI: − 0.35, 0.07; *p* = 0.19) and *g*_b_ =  − 0.16 (95% CI: − 0.36, 0.05; *p* = 0.14). Of the six studies, only one [18] reported significant short-term (3 months) and long-term (20 months) intervention effects of reduced depression symptoms in parents (see Figs. S17, S18, S23 and S24). Overall, the findings suggest small-to-no treatment effects for parent mental health outcomes. Heterogeneity levels were small-to-medium across all analyses (see Figs. S17–S24 in supplement materials for all time-points and sensitivity analyses).

#### Bias assessment

Potential publication biases were assessed visually with the help of funnel plots (Figs. S25–S27). The funnel plots do not suggest an asymmetry; however, the number of studies included was small, thereby increasing the likelihood of any deviation or adherence to the funnel shape being by chance. The funnel plots do suggest that studies are missing at the top and bottom for both significant and non-significant areas, which highlights a gap for studies involving larger sample sizes. The performed Egger’s tests were non-significant, thus indicating no bias due to small-study effects. However, funnel plots are influenced by multiple factors, of which publication bias is only one. Poor methodological quality and between-study heterogeneity could both influence the funnel plot in this review case. The conducted Galbraith plots suggest that two studies [19, 20] may have influenced the pooled effect size in the meta-analyses towards a greater reduction of child internalising symptoms (see Figs. S28–S32).

#### Meta-regressions

Meta-regressions were only performed for child and parent outcomes when sufficient studies were available. We included quality rating, type of control group and number of sessions as predictor variables, and mental health outcomes at first follow-up as the outcome. None of the meta-regressions suggested any significant effects for the included predictor variables. See Supplement materials 1 for results of meta-regression.

### Synthesis of intervention experience and acceptability findings

#### Description of studies

Twenty-two studies reported on families’ experiences with the interventions, the perceived benefits, and intervention acceptability. Ten of these studies, reporting on eight different interventions, described families’ experiences using qualitative methods. Together, these studies included 320 family members, of which 179 were parents/carers, 135 were children or young people, and 13 were former service users. Three papers included facilitators (*n* = 62) and/or referrers (*n* = 5). Four interventions targeted depression, one substance abuse, one anxiety, and three were open to multiple parental mental health illnesses. The majority of the interventions (62%; 5/8) were group interventions, providing 6–12 sessions, except for The Family Model providing only one session, and KidsTime being open-ended. Fourteen studies described acceptability in a quantitative manner, sharing experiences of at least 372 families (two studies did not provide sample size on family level), participating in 12 different interventions. Two studies (ID-*n*: 143, 173) provided attendance data, reporting attendance levels of at least 70% (of sessions/participants/active engagement), although there was no uniform way to assess attendance. One study evaluated an app-enhanced intervention (ID-*n* = 5), where engagement with the app was reported to be around 50% (of the days).

#### Description of themes

We derived three themes that describe families’ perceived benefits and outcomes of taking part and related intervention change mechanisms. In terms of intervention acceptability and families’ experiences of taking part, we summarised the evidence in four themes (Table 5).

**Table 5:** Themes, descriptions, and quotes

Theme	Description	Quote
***Topic 1: Perceived benefits outcomes and change mechanisms***		
**1. Learning, knowledge, and skill development**		
1.1 Increased knowledge and understanding of mental illness	Psychoeducation and mental health literacy components of interventions helped increase parent and children’s knowledge of mental health problems affecting their family and the potential impacts of mental illness on the family. This led to increased understanding of family member’s experiences of PMI. In group settings, the sharing of experiences and advice also provided additional knowledge and understanding	“I wanted him to be able to have an understanding... the signs of what to look out for, not even in myself, but say for his peers, amongst him and what have you and even himself... I was willing to give it a go and what have you, for the simple reasons, I wanted [my child] to have a bit of an understanding as to why I can be like the way I am.” (W16P16A1, ID-*n*:10)
1.2 Coping skills development	Both parents and children reported developing coping skills in how to deal with the impacts of PMI. This included general coping skills, increasing well-being and self-esteem and for children developing ways of dealing with school problems (e.g. concentration). Children also reported learning who to turn to when they needed support with PMI and learning how to respond to parental mental health problems	“When we know she’s becoming ill, we kind of bring up strategies and things, you do this, you do that, and if you don’t do this she’s going to … you know, we have to talk about it, yes, definitely. So we’re both happy to talk about it, but talking about it to my mum.” (Young person past attendee, ID-*n*: 59) “I can’t really change [my parent], I have to learn to live with that... getting on with my life and not letting [my parent’s] problems affect me…I don’t want it to get me down, I’ve got a sense that I can get on with my life without letting it worry me.” (Child, ID-*n*: 79)
1.3 Parenting skills	Change in parenting skills and confidence in skills Parents reported learning practical parenting skills to support their interactions with their child and feel more confident in their parenting skills	“Being able to try out such a reward system for a bit, or simply implementing certain parenting strategies that I’d heard of before in theory but just never really officially tried out." (Parent of family 5, ID: 16)
**2. Enhanced family environment and relationships**		
2.1 Family relationships	Interventions improved family bonding, relationships (parent–child, couple and sibling) and cohesion at home. Shared activities and sharing experiences in the interventions led to more of these activities at home as well. This was seen to lead to healthier family dynamics and a shift in roles and responsibilities	“I have a better understanding of how my mum feels... [which has made their relationship] a lot better because we’re getting along a lot better now... We’re not arguing anymore.” (W01Y02A1, ID-*n*: 10) “... it brought us closer together so we can bond... a bit more, cos normally I’m out just playing with my mates and [my brother’s] out with his mates and mum’s in her room, now mum comes out of her room we all sit down downstairs, watch TV, eat dinner together, which we never used to do before.” (Child; ID-*n*: 79)
2.2 Communication	There were mixed responses about how interventions had improved family communication. For some, communication had improved (around PMI and in general), reducing the level of conflict in the home. Others did not perceive any changes in communication about PMI in the home due to it being challenging to maintain the communication skills learnt during intervention sessions	“to be calm... to actually listen, I know I listen to what the kids say... but I’d learned to actually listen properly to what they were saying not just the bits and pieces I wanted to hear... I think it’s really helped me.” (Adult; ID:79) "Simply the, uh, distance to the topic was kind of gone afterwards and that helps of course, yes, I have to admit that helped me an incredible amount." (Parent of family 8, ID-*n*: 16) “How much should we talk to him about this at all? It feels difficult […] if we are going to talk more about it then we need some more help to know how to raise the issue.” (Henny, ID-*n*: 2)
**3. Normalisation and like-mindedness**		
3.1 Reducing shame and guilt, feeling normal	For those that took part in group interventions, parents reported feeling less alone in their experiences. These social connections, but also the psychoeducation provided, helped families feel less guilt and shame around their mental illness	“I felt quite lucky and I’d sometimes walk into the group feeling really down and really sad and really alone and really isolated and then I’d look at the group and think, hang on, yes, I’m like this but my life is really not that bad... it’s good to know that people are in the same boat as you... It’s made me feel less guilty.” (parent, ID-*n*: 10) “It does help because…I feel a bit normal, sometimes I do not feel good, I’m strange, I’m different it makes me feel normal and it makes [my child] more relaxed…..it makes us more relaxed about me, because when as well when [my child] just started to realize that I have some problems they were very scared because it is scary if you don’t know what you deal with and somehow they put it in the normal perspective, normal like sort of it’s okay.” (Parent current attendee, ID-*n* 59)
***Topic 2: Intervention acceptability and families’ experiences of taking part***		
**4. Initial engagement**	Families were reluctant to participate for a variety of reasons, including • Feeling guilt and shame • A lack of understanding of the intervention • Being nervous of the unknown, or the group • Reluctancy to engage other family members (e.g., worry about the impact of talking about PMI with children)	“I felt shy, I wondered what it was going to do, what it was about. Now I know. It is about having fun and mental illness so when mum or dad get ill, you can help them.” (Young attendee, ID-*n* 214)
**5. Role of facilitators**		
5.1 Engagement	Facilitators were perceived to have an important role in engaging families in the interventions. This included meeting people before the interventions began and creating a safe and non-judgemental space. Facilitators made the confidentiality and privacy of the sessions clear, as well as being welcoming and friendly to create a comfortable atmosphere for families. One study raised that having consistent facilitators (i.e. low staff turnover) was also important	Families required extensive preparatory input from clinicians to allay their fears to persuade them to engage with FT. (ID-*n* 206) The parents experienced the intervention meetings as a safe and non-judgemental context for them and their family. These feelings helped them to speak openly about things that could be painful. (ID 2) I think [Young SMILES facilitator] was lovely. I think her approach... her happy, bubbly personality, I think really took off in the group. She was very easy to talk to and any issues I’ve had outside the group, she’s been fantastic and supportive. I think that’s really good. (ID 10)
5.2 Skills	Skills of the facilitator were also deemed important and related to managing groups, providing child-centred care (for children of different ages), having appropriate knowledge and expertise, managing different perspectives, and engaging families	The majority of PMIs and partners indicated that it was the skill of FT clinicians that mediated the benefits for families. (ID-*n*: 206)
**6. Content**		
6.1 Focus of topics	Most families were happy with the topics being discussed (see benefits). For some, the focus was too narrow or slightly misplaced (e.g., too much on child or parent, or not enough on problems of other family members). In some intervention, intervention-specific topics were deemed helpful by families, such as the focus on culture and immigration in Family Strength (ID 91). Other elements were perceived with mixed feelings, such as the joint parent–child exposure in the Enhanced transdiagnostic CBT (ID 213)	“It was directed towards me as I’m the one who is ill. Sometimes I felt that everything was my fault because I’m ill, as if ‘you’re ill and therefore you’re the problem and it’s you we have to fix.” (Parent ID-*n*:2) Parents endorsed both positive and negative experienced or anticipated emotions in response to the observational exposures, suggesting a complex or mixed feeling state. (ID-*n*: 213)
6.2 Talking about PMI	Many interventions facilitated in-session talk about PMI. Opening up and discussing things in sessions could be emotionally challenging for some, due to fear of disclosure or a lack of confidentiality. There were fears about how others in a group setting may respond	It became apparent that, for some CYP, a major barrier to talking was the expectation that their thoughts and feelings would be shared with their parents. (ID-*n*: 10) The findings suggest that FT was challenging for many families despite the non-judgemental support provided by FT clinicians. Several parents/partners reported difficulties in speaking openly in sessions and/or listening to the experiences of family members. (ID-*n*: 206)
**7. Intervention format, structure, and logistics**		
7.1 Length and intensity	A recurring theme was the need for more or longer support. Families wanted more time to discuss the topics or do the exercises of the interventions. This was sometimes because they felt the intervention was rushed or crammed, or because it took time to feel enough trust to fully participate. Some families wanted to continue their meetings with the other families to continue to build on the social connections made. For some families, the interventions were too intensive, for example because the assessment was invasive, homework was too much, or because it was time-consuming	“I think more sessions with the family... and more time with the children would have really helped. The three of them went in one by one for 20 min. So it might have been a little bit rushed for them, they might not have had enough time.” (PMI 16. ID-*n*: 206) “When we finished the programme I didn’t have no-one else to talk to and I wanted to see [the workers] to speak to them a bit more... I felt quite sad because you had just go to know all the workers there and all the kids there but then you wouldn’t get to see them no more.” (Child, ID-*n*: 79) The most commonly named disadvantage to participating in the intervention was how tiring and time-consuming it was (*N* = 17) due to weekly sessions and homework assignments. (ID-*n*:16)
7.2 Group approach	Both adults and children talked about the benefits of connecting with other families in similar circumstances and share experiences Being together provided a safe space for some to discuss things they might not discuss with anyone else. Whereas for others, the group felt unsafe, they were nervous to share information, or they had unpleasant experiences with other group members Individual characteristics also impacted the acceptability of group approach, including having a different language, children with a disability, and the age range of children. Wide age ranges made it hard to accommodate the needs of all children. Some older children felt as an outsider or did not enjoy it, because they did not have anyone of their age	“I felt quite lucky and I’d sometimes walk into the group feeling really down and really sad and really alone and really isolated and then I’d look at the group and think, hang on, yes, I’m like this but my life is really not that bad... it’s good to know that people are in the same boat as you... It’s made me feel less guilty.” (parent, ID-*n*: 10) “The open group talks allowed us, including myself, to really open up and speak about things I couldn’t imagine talking about with any of my friends.” (Child, ID-n: 79) Disappointment by lack of meaningful conversations about personal experiences left some parents feeling isolated, like ‘a stranger at a bus stop’ (N29P01A1), whereas others felt frustration with their experience of being in a group with parents who did not contribute. (ID-*n*: 10) “Well, basically, like I just asked them how they figure out their problems and they tell me and I just tell them how I do it.” (ID-*n*: 10)
7.3 Interactive activities	Interactive and dynamic activities were perceived as helpful and enjoyable by families, for example drama and role-play. Having fun and playing (with friends) was deemed important, especially for children. It provided a welcome break of the hardship at home	“Home was sad, Kidstime was fun. That’s what I looked forward to. I looked forward to having fun, you know being a child. But at home you have to be an adult, look after yourself, look after mum, look after the house, give her medication; at Kidstime you’re having fun. You’re being looked after and you’re not looking after others. …there are people there who are paying attention to you and you can go and speak to because you probably can’t speak to your mum because you know she’s not well she probably won’t understand. But Kidstime was time for the kids; I think that’s why it’s called Kidstime.” (Young person past attendee, ID-*n*: 59)
7.4 Manual	There were contrasting views about manuals in the interventions. In Family Talk (ID2), the manual and the structure that was provided was seen as a positive. Whereas families and facilitators in Young Smiles (ID 10) reflected on the need for the sessions to have flexibility to meet the individual needs of families	Some emphasised that they appreciated the structure of the intervention and that the care providers followed a manual. One parent expressed that the structure made her feel safe and secure, another parent expressed that it could have been ‘messy’ without a manual. (ID-*n*: 2) Facilitators’ abilities and efforts in adapting to individual circumstances were key to the acceptability of the intervention and continued participation. Providing individual support when required was valued. (ID-*n*:10)
7.5 Setting and environment	The location and time of the interventions impacted acceptability. Participation was sometimes difficult due to accessibility issues and facilitators had to support organising transport	Engagement could also be compromised by issues in terms of organisation of transport to the workshops. (ID-*n*: 59)
7.6 Whole-family component	The family component of interventions allowed for many of the benefits listed above (having fun together, shared understanding, listening to each other). Some families mentioned that they would have liked additional family members or even peers to have joined in the intervention. Some families said they would have wanted more family time during sessions. In Young Smiles, lack of parental engagement was observed to relate to worse outcomes. Nevertheless, some families described that separate sessions could also be beneficial, providing a space of respite for the children	“Some parents highlighted that they found it particularly valuable to listen to children speak about their experiences and feelings, with some of the facilitators emphasising the importance of giving children a voice.” (ID-*n*: 79) “It was kind of good because mum wouldn’t be there and she wouldn’t be seeing the stuff that we said. Not saying that in a bad way but like, she would just go ‘don’t say that’.” (ID 10) A small number of cases where participants wished that other members of their family, such as younger children or misusing parents, could attend. (ID-*n*: 79) “Because you get to spend time with them and I don’t really do that.” (ID-*n*: 10)

##### Topic 1: Perceived benefits, outcomes, and change mechanisms

Findings from the qualitative studies suggest that most families reported feeling positive about the outcomes of the interventions they had received and described it as a helpful experience. In some studies (ID-*n*: 2, 15, 214), participants described not noticing specific changes or improvements in response to the intervention (“*To be honest no, I don’t think it made any great difference in the long run*” parent, ID-*n*: 2). Two studies assessed whether any harm was experienced by participating families, and nothing was reported.

*Theme 1: Learning, understanding, and skill development.* Studies commonly highlighted that interventions enhanced participants’ levels of understanding, knowledge, and skills. Families reported that receiving practical information (e.g., who to call when, how to structure a day) and psychoeducation (general and specific to PMI) not only increased their knowledge and mental health literacy but also helped them feel more confident (ID-*n*: 2, 10, 16, 59, 79, 91, and 214). Sharing experiences with other families and family members contributed to a better understanding of different perspectives (e.g., the impact of PMI on children and partner) and supported mutual learning sometimes by exchanging practical advice (ID-*n*: 10, 79, 214). Some studies described families’ mentioning intervention-specific outcomes, such as children reporting that they had learned new coping and problem-solving skills which helped them with reducing stress, anxiety and worries (ID-*n*: 16, 91). Many interventions aimed to improve parenting skills, by providing feedback, support, and advice around parenting. Parents in these interventions primarily reported to have benefited from the support and feeling more confident as parents (ID-*n*: 2, 16, 206). Some parents wished for more ongoing support as their children are getting older (ID-*n*: 2). Changes in parenting as reported by parents were not always noticed or reported by their participating children (ID-*n*:2).

*Theme 2: Enhanced family environments and relationships.* Many families reported that interventions had created more warmth in their families and increased bonding between family members, including parent–child, couple, and sibling relationships. Exercises that encouraged sharing of experiences and perspective-taking between family members were described as bringing family members closer together by making sure everyone’s voice is heard and validating different experiences. Interventions that involved activities for the whole family (e.g., fun activities, talking about strengths) were perceived to increase families’ confidence and trust. Most families described that they enjoyed spending time together as part of the intervention. For some families, this naturally led to more engagement in family activities outside of the programme. Parents also noted that building a “united front” helped them be better parents. Some interventions were described as contributing to healthier family dynamics, by helping with the shift in roles and responsibilities (e.g., children having less responsibility).

Many families described being more able to talk about PMI in their family, and that interventions had helped parents by finding age-appropriate words to talk about mental illness and families by developing a shared language for these conversions. However, talking about PMI as part of the intervention was experienced as challenging by some families (ID 206). Occasionally, families reported that they did not notice any changes in the way they spoke about PMI in comparison to prior intervention. Families also noticed general improvements in communication within the family and explained that they had learned to listen better and respect each other, which in turn led to fewer conflicts, better problem-solving, and increased understanding and support for each other (ID 10, 79, 91, 206).

*Theme 3: Normalisation and like-mindedness.* Meeting other families and peers in interventions and having the opportunity to share experiences was associated with reduced feelings of stigma, guilt, and shame. Families explained that hearing similar stories helped them feel more normal (ID: 10, 16, 59, 79, 91, 206, 213, and 214). Many young people and adults also shared that they benefited from making new friends and meeting families who lived in similar circumstances, which made them feel less alone. It also helped them feel more comfortable and safer around them, as opposed to friends and peers that they had elsewhere, for example at school (ID-*n*: 10, 59, 79, 214). These benefits were primarily reported in interventions with peer or group components.

##### Topic 2: Intervention acceptability and families’ experiences of taking part

Studies that assessed acceptability and satisfaction rates via questionnaires mostly reported high satisfaction scores (ID-*n*: 5, 62, 91, 118, 123, 132, 135, 201, 179, and 214). When questionnaires were specific enough, family members tended to rate the support and information received by facilitators the highest, and homework assignments or exercises somewhat lower on satisfaction scales. The only exception was Family Strengths (ID-*n* = 91), where participants especially appreciated the family exercises (e.g., family fun time). The five themes below summarise the qualitative findings from ten studies.

*Theme 4: Initial engagement.* Studies that explored families’ reasons, motivation, and expectations to take part described that some families had been unclear about the purpose of the intervention, but that most parents had hoped to support their children better. Due to the limited understanding, families could not always provide clear reasons for attending but explained in many cases that the intervention was the only support offered to them. The uncertainty about the intervention and lack of information about what it would entail resulted in families feeling initial apprehensions about taking part. Many participants reported feeling anxious and nervous at the beginning of the intervention but that this had eased over time.

*Theme 5: Role of facilitators.* Facilitators were often mentioned as important drivers for engaging families in and for the acceptability of the intervention. Almost all studies talked about the facilitators being welcoming, non-judgemental, and following a strength-based approach. Several studies provided positive feedback on the flexibility of facilitators and their ability to adapt to individual circumstances. For children, the fun and welcoming atmosphere created by facilitators was important for satisfaction and engagement with the intervention. In one study (ID-10), parents shared their negative experiences with the facilitating team and described initial meetings and assessments as invasive and not family-centred. Parents in the same study reported that facilitators had been overinvolved, calling children’s schools and putting too much pressure on participating parents.

*Theme 6: Intervention content.* Families’ satisfaction with the content of interventions varied. Most families gave positive feedback about intervention content and reported that it had contributed to the perceived benefits (e.g., learning) and positive changes. Families also provided suggestions on how interventions could be further improved. One study reported that the impact of PMI had not been addressed enough, while some studies indicated that (Young Smiles, Family Strengths, and Family Talk) that parents had found that the intervention had focused too narrowly on PMI and/or the impact it had on the children, which occasionally made the affected parent feel uncomfortable and that they were the “cause” of the problem or the one to blame. Some families also explained that they wished for more wider issues and concerns to be addressed as their and their children’s mental health and well-being were impacted by other factors unrelated to PMI (e.g., housing, and physical health problems). Interventions with group components were often criticised as not being suitable or engaging enough for different age groups, specifically psychoeducation and activities, and inaccessible for individuals with disabilities (ID-*n*: 59, 214). Families who took part in interventions that included playful and/or creative activities experienced these as helpful in terms of practising and exploring new skills, but also described them as fun and enjoyable which had helped them feel more positive generally and also by giving them time away from home (*“Home was sad, Kidstime was fun. That’s what I looked forward to. I looked forward to having fun, you know being a child. But at home you have to be an adult, look after yourself, look after mum*”—child ID-*n*: 59).

*Theme 7: Intervention format, structure, and logistics.* As mentioned above, interventions with a group format were generally associated with many positive experiences by families, including meeting other families, sharing experiences, feeling less socially isolated, and learning from others. In some studies, parents reported that the group size had been too big, which had made them feel stressed and in some cases also led to discontinuation with the programme. The group format was also described as being anxiety and shame provoking when having to share personal experiences with new people. At times they could also feel overwhelming and unsafe. In one study, parents shared their frustration about in-active participants and participants behaving unprofessionally (ID-10). Participants repeatedly emphasised the importance of having sufficient time to “settle in” to feel safe and build trust with other participants. Families also described that these concerns were more easily overcome in groups that were informal and felt non-judgemental and welcoming.

Most interventions followed a regular structure with weekly or biweekly meetings. Families reported that they appreciated a regular structure and manualized approach, but occasionally families described having to attend weekly meetings and doing homework exercises could be challenging and tiring. They wished for more flexibility to meet families’ needs. Most of the interventions evaluated were closed-ended interventions, with a fixed number of sessions. Many families shared that they needed more sessions to sustain and implement the achieved changes and that they hoped for more continuous support, as in many cases no other support was available once this specific intervention had finished. Some parents explained that they wanted more support as their children got older and some families simply wished to keep in touch with other group members to maintain their new social support network.

Families considered the environment where the intervention took place as important and commented on certain settings and locations pointing out that some felt more welcoming (e.g., community centre) than others (e.g., clinic, small rooms). Occasionally, it was reported that locations were hard to reach for families which could impact attendance and engagement in the programme.

Interventions providing mainly family sessions were praised for their whole-family approach, whereas participants from other interventions requested to include more family members (ID 16) or to have more whole-family sessions rather than separate parent and child sessions only. In one study (ID91), parents wished for more adult time to work on their marital relationship. For young people, the data suggested that adolescents preferred adolescent-only over the parent–child sessions, based on higher satisfaction and alliance ratings (ID-*n* = 62) and young people explaining that the child-only sessions provided space where they could be autonomous from their parents and which provided some respite (ID-*n*: 10).

## Discussion

This systematic review and meta-analysis identified 66 reports from 41 independent studies that evaluated 30 different whole-family interventions focussed on supporting families affected by parental mental illness. Researchers and practitioners have long emphasised the need for whole-family approaches and continuing evidence gap [21, 22]; therefore, in contrast to previous reviews [3], we exclusively looked at the evidence for interventions that target the whole family, which included both children and parents/caregivers. Additionally, we have summarised families’ experiences with and acceptance of these interventions, which has not been present in previous reviews [7].

The results of the meta-analysis and quantitative synthesis indicated a need for higher quality research and evidence to draw clear conclusions on the effectiveness of whole-family interventions. In relation to children’s internalising problems, the meta-analysis (*t* = 12) suggested small-effect sizes, which was confirmed in the quantitative synthesis (*t* = 27) where studies with higher quality ratings consistently reported small-to-medium effects. These findings are similar to reviews of child-only interventions aimed at reducing the risk of mental illness in children of parent mental illness [7]. The impact of existing interventions on externalising symptoms was less often assessed and the quality of studies was lower (i.e., small sample sizes, no descriptive statistics reported). For parent mental health outcomes, most studies reported positive outcomes; however, only half of these studies reported any effect sizes, which albeit moderate-to-large, were from studies with low-quality ratings. Findings from the meta-analysis (*t* = 6) indicated small-to-null effects in terms of interventions’ effectiveness to reduce parental mental health difficulties. However, it is important to note that less than half of the interventions we reviewed included a component specially to address the parent mental illness symptomatology. The findings from the meta-analysis may be explained by whole-family interventions having a greater focus on supporting families to learn to live with mental illness in the family, rather than treating symptoms of mental illness.

Most of the whole-family interventions identified had a core component of improving communication within families, psychoeducation to enhance understanding of mental illness, developing parenting skills and coping skills for both generations. There was great variety in the use of measures to assess family-related outcomes in the publication reviewed, with many studies not employing standardised measures and only two studies reporting effect sizes. The quantitative findings indicated that fewer than half of the studies reported positive changes in response to the intervention. However, the qualitative synthesis indicated that families did report improvements in family-related outcomes, such as better communication and understanding of the experiences of parent mental illness, and increased time spent together in positive interactions. In particular, psychoeducation components were perceived as being helpful. In line with other research, the qualitative findings suggest that mental health literacy delivered with the additional context of the family experience is particularly helpful for families [23, 24]. Given family-related outcomes were a key aim of many of the interventions, future research focussing on whole-family interventions needs to ensure that these dimensions are properly assessed, especially considering the evidence that family functioning and good parenting are protective factors for both parents and children.

All interventions followed a structured approach with regular sessions, whereby the majority provided a fixed number of sessions, while two programmes were open-end. The quantitative findings indicated that intervention effectiveness tended to decrease with longer follow-up times, suggesting that families may need more ongoing support; perhaps, in the form of subsequent booster sessions, future research and interventions should consider this and explore this with families. The qualitative findings also highlighted that many families felt left without any support once fixed term sessions had ended and concerns were raised about accessing ongoing support as children and young people age and families go through life transitions. One way to address this may be through additional programmes such as ‘The Think Family-Whole Family Programme’ [25] or the ‘CAMILLE training programme’ [26] which aim to train professionals to raise awareness of the incidence, context, and impact of parent mental illness. Programmes like these, that help professionals have the skills and confidence to address the needs of families with parent mental illness, alongside specific whole-family interventions for families may help continue the effects and support to families over the lifespan.

About half of the interventions included multi-family or group components and one-third focused on improving families’ social support networks, which may also help with the continued support families need and want. The collated evidence from this review suggests that most families experience group interventions as positive, highlighting specific components, such as meeting other families, sharing experiences, and establishing social connections helping to reduce social isolation and help normalise their experiences. The current evidence regarding the effectiveness of group interventions [27–29] and peer-support programmes [30] in preventing and reducing psychological symptoms has been mixed. However, a recent study showed that group cognitive behavioural therapy can help reduce stigma [31] and it has been emphasised by others [32] that peer-support programmes should be seen as complementing clinical interventions, as they provide a different type of practical, social and community support.

Going forward, it would be helpful to map out how different components across clinical and non-clinical (i.e. prevention and maintenance) programmes can be utilised, separately and combined, to address a wider range of target outcomes (beyond clinical symptoms) that are relevant to families with parental mental illness.

### Clinical implications

It is essential to support families living with PMI and whole-family interventions provide an opportunity to mitigate potential negative outcomes as well as ameliorating existing difficulties. There is still no theoretical consensus as to the most important mechanism to improve outcomes for these families in general and also more specifically considering different family characteristics or even time-points in their journey [e.g. a parent being (un-)diagnosed, parent in hospital]. It is essential that clinical practise is rooted in theory, and therefore, more research must be conducted on the mechanisms of effectiveness in whole-family interventions, and families with lived experience of mental health must be consulted. This review provides an important overview of the different intervention types and components, their aims and mechanisms, which can guide researchers and professionals in getting a better understanding of the types of support available and how we can align them with families’ needs.

More large-scale randomised-controlled trials are needed before it can be stated what type of intervention would be most beneficial to families in clinical practise. It is promising that there are currently larger trials being undertaken such as the VIA family, a whole-family multicomponent intervention for families where a parent has psychosis or bipolar [33]. In the meantime, clinicians must continue to ask adult service users about the presence of children as well as their experiences of parenting and consider the systemic implications of mental health. We are aware that, despite many positive attitudes in families and practitioners, structural barriers exist for bringing child and adult mental health services closer together to enable whole-family approaches, and hope that research like this can help overcome some of these barriers.

### Strength and limitations

The present review fills an important gap in the literature by summarising the evidence for whole-family interventions to support families living with parental mental illness and highlighting where more work is needed. It investigates families’ experiences with these interventions, which has previously been neglected in the literature. Our findings provide an overview of the current evidence landscape and in relation to that there are a few limitations that need to be considered when interpreting our findings.

The level of quality and information provided in primary research significantly determines the quality of systematic reviews. There was a significant lack of high-quality trials, many being limited by insufficient sample sizes, absence of a control group or lack of providing relevant descriptive statistics, and effect sizes. Additionally, only very few studies include sufficient long-term follow-up assessments which limits insights regarding programs’ long-term effects. In relation to that, many studies with a prevention focus report and assess changes of mental ill-health, instead of incidence rates of disorder onset or other prevention outcomes, such as quality of life. Furthermore, quality ratings had to be based on the information provided by authors, which led to different quality ratings for the same study. Thus, quality ratings provided here may not reflect the full quality of each study. In relation to that, many intervention descriptions are often not detailed enough, or intervention manuals are not provided/accessible, thereby highlighting a need for researchers and practitioners to be more transparent and provide more detail of the interventions.

Due to the lack of studies reporting correlations between measures and within assessment time-points, we were unable to conduct a multi-level meta-analysis, which would have allowed us to better explore within-study variation. Therefore, the mean effect sizes presented were estimated across multiple separate meta-analyses.

Our definition of “whole-family interventions” allowed us to include a wide range of interventions, and therefore, whole-family components varied significantly across studies, with some interventions offering 12 sessions to the whole family, others only offering two sessions and other interventions only included assignments for families to do at home. Hence, more research is needed to get a better understanding of what whole-family approaches are most suitable and beneficial.

## Conclusion

Evidence has suggested that researchers and practitioners have neglected whole-family intervention approaches, even though they are expected to be more beneficial than child- or parent-focused interventions alone [2, 3]. Our systematic review shows that the existing interventions seem to have small effects on child mental health and family outcomes and that many families have reported positive experiences with these interventions. Despite the promising nature of whole-family interventions, the evidence base is still in its infancy. Our findings highlight that more high-quality research needs to be conducted and that there is a lot of untapped potential for whole-family interventions. We recommend that families with PMI are more closely involved in the further development of these interventions to enhance their potential as well as their evaluation, so that researchers also capture what matters to families.

## Supplementary Information

Below is the link to the electronic supplementary material.

Supplementary file1 (XLSX 109 KB)

Supplementary file2 (XLSX 146 KB)

Supplementary file3 (DOCX 3307 KB)

## Data Availability

All extracted data and meta-data that were created as part of this study can be accessed via the Open Science Framework [9].
