# Supplementary material for: Whole-family programmes for families living with parental mental illness: a systematic review and meta-analysis
Source: Eur Child Adolesc Psychiatry. 2024 Feb 23;33(9):3203–46. doi: 10.1007/s00787-024-02380-3 (PMC11424744; doi:10.1007/s00787-024-02380-3)
Supplement: Supplementary file 3 — Supplementary file3 (DOCX 3307 KB) [file 787_2024_2380_MOESM3_ESM.docx]

# Supplementary materials

The material below accompanies the following manuscript: Whole-family intervention programmes for families impacted by parental mental illness: a systematic scoping review and meta-analysis. Submitted to the Journal of European Child and Adolescent Psychiatry. Authors: Bettina Moltrecht*, Aurelie M. C. Lange, Hannah Merrick, Jessica Radley

* Corresponding author: b.moltrecht@ucl.ac.uk

Contents

[Supplementary materials 1](#_Toc150158584)

[Sample characteristics 2](#_Toc150158585)

[Overview of papers included in review with assigned IDs for trial (ID-t) and paper (ID-n) 6](#_Toc150158586)

[Search Strategy 13](#_Toc150158587)

[Intervention components 14](#_Toc150158588)

[Definition of coded intervention components 15](#_Toc150158589)

[Meta-analyses results 16](#_Toc150158590)

[Child internalising outcomes reported by child 16](#_Toc150158591)

[Child internalising outcomes reported by parents 21](#_Toc150158592)

[Parent mental health outcomes 26](#_Toc150158593)

[Meta-regressions 30](#_Toc150158594)

[Meta-regression with child internalising outcomes reported by children at first follow-up 30](#_Toc150158595)

[Meta-regression with child internalising outcomes reported by children at second follow-up 30](#_Toc150158596)

[Meta-regression with child internalising outcomes reported by parents at first follow-up 31](#_Toc150158597)

[Meta-regression with child internalising outcomes reported by parents at second follow-up 31](#_Toc150158598)

[Meta-regression with parent outcomes reported at first follow-up 32](#_Toc150158599)

[Effect sizes across follow-up time 32](#_Toc150158600)

[Effect sizes of child internalising problems per study and follow-up 32](#_Toc150158601)

[Effect sizes of parent internalising problems per study and follow-up 33](#_Toc150158602)

[Bias assessment 34](#_Toc150158603)

[Funnel plots 34](#_Toc150158604)

[Galbraith plots 36](#_Toc150158605)

[Egger’s tests: 38](#_Toc150158606)

[Trials excluded from meta-analysis with reasons 40](#_Toc150158607)

## Sample characteristics

Table 1S. Sample characteristics per study

| **Study ID** | **Report ID** | **Country** | **N families** | **Age children M(SD)** | **Age parent M(SD)** | **Female gender children** | **Female gender parents** | **Ethnicity** |
| --- | --- | --- | --- | --- | --- | --- | --- | --- |
| 1 | 1 | Greece | N_t_ 30  N_c_ 32 | _t_ 11.7 (2.6)  _c_ 12.3 (2.7) | _t_ 41.4 (5.6)  _c_ 41.1 (5.4) | _t_ 53%  _c_ 40.6% | _t_ 80%  _c_ 81.3% | not stated |
| 2 | 73 | GER | N_t_ 28  N_c_ 9 | 10.4 (2.7) | not stated | 63.6% | not stated | not stated |
| 3 | 93  119  123 | Finland | N_t_ 53  N_c_ 56 | 11.9 (2.6) | not stated | not stated | _t_60.0%  _c_59.3% | not stated |
| 4 | 186 | USA | N_t_ 7 | 10.9 (2.0) | not stated | not stated | not stated | not stated |
| 5 | 180 | USA | N_t+c_ 28 | 10.5 (-) | 42.9 (4.8) | not stated | not stated | not stated |
| 6 | 148  162  177  178  179 | USA | N_t_ 55  N_c_ 44 | _t_11.6 (1.9)  _c_11.5 (2.03) | 43.1 | 42.7% | 75.7% | not stated |
| 7 | 5 | USA | N_t_ 22 | 15.4 (1.8) | not stated | 45.5% | 61.8% | ***Children***  White 68.2%  African American 9.1%  Mixed ethnicity 22.7% |
| 8 | 8  20  34  70 | USA | N_t_ 70  N_c_ 66 | _t_ 8.5 (1.8)  _c_ 8.9 (1.9) | _t_ 39.9 (4.8)  _c_ 41.8 (5.1) | _t_ 62.9%  _c_ 48.5% | _t_ 77.1%  _c_ 80.3% | ***Children***  Caucasian: _t_82.9%, _c_ 86.4% |
| 9 | 132 | USA | N_t_ 20  N_c_20 | _t_ 9.2 (1.9)  _c_ 8.7 (1.8) | _t_ 41.4 (7.4)  _c_ 40.7 (4.3) | _t_ 50%  _c_ 40% | not stated | ***Children***  Caucasian _t_100%, _c_ 80% |
| 10 | 10 | UK | N_t_ 18  N_c_ 15 | _t_ 10.2 (2.1)  _c_ 10.9 (2.7) | Range 25-64 | _t_ 56%  _c_ 60% | _t_ 94%  _c_ 87% | ***Parents***  White: _t_ 89%, _c_93% |
| 11 | 11  19  31  48 | USA | N_t_ 123  N_c_ 60 | 11.5 (-) | 33.9 (-) | 48.1% | 100% | ***Parent***  Hispanic 53.6%  African American 42.6%  Other 3.8% |
| 12 | 21 | USA | N_t_ 49  N_c_ 19 | not stated | 33.8 (-) | not stated | 100% | ***Parent***  White 60.3% |
| 13 | 201  16 | GER | N_t_ 50  N_c_ 50 | _t_ 11.7 (2.8)  _c_ 12.0 (2.9) | _t_ 45.2 (5.8)  _c_ 47.1 (7.0) | _t_ 55.1%  _c_ 52% | _t_ 60%  _c_ 62.7% | ***Families***  German nationals (91.5%)  Turkish, Croatian, Bulgarian, Austrian (8.5%) |
| 14 | 18  29  72  82  105  116  122  138 | USA | N_t_ 90  N_c_ 90 | 11.5 (2.0) | Mother 41.16 (7.17)  Father - 48.3 (7.50) | 50% | 88.9% | ***Parent***  82% Euro-American, 12% African American, 2% Hispanic American,  1% Asian American,  1% Native American,  2% mixed ethnicity.  ***Child***  74% Euro-American, 13% African American,  3% Asian American,  2% Hispanic American,  1% Native American,  7% mixed ethnicity. |
| 15 | 26 | GER | N_t_ 41  N_c_ 26 | _t_ 10.1 (2.3)  _c_ 10.3 (2.7) | _t_ 40.0 (6.6)  _c_ 39.3 (5.5) | 51.0% | 73.2% | not reported |
| 16 | 45 | Canada | N_t_ 19 | 9.7 (2.6) | 43.3 (8.5) | 54% | 69% | not reported |
| 17 | 62 | USA | N_t_ 16  N_c_ 8 | _t_ 14.7 (1.8)  _c_ 14.0 (1.7) | _t_ 44.9 (7.5)  _c_ 42.3 (8.9) | _t_ 87.5%  _c_ 75.0% | _t_ 93.8%  _c_ 100% | not reported |
| 18 | 67 | AUS | N_t_ 89 | 10.4 (2.4) | not reported | 57.3% | not reported | not reported |
| 19 | 71 | UK | N_t1_ 69  N_c_ 71 | 6-13 years | not reported | _t1_52.1%  _c_54.9% | 100% | ***Child***  White British: _t_94.4%, _c_ 77.5% |
| 21 | 91 | USA | N_t_ 13 | 9-18 years | 35.6 (4.3) | not reported | 100% | Latin American 100% |
| 22 | 101 | USA | N_t_ 16  N_c_ 14 | 12-15 years | not reported | _t_ 44%  _c_ 43% | _t_ 88%  _c_ 93% | ***Parent***  Mixed ethnicities: _t_ 38%; _c_ 31%  Caucasian: _t_ 63%, _c_ 69%  Hispanic: _t_ 19%, _c_ 23%  ***Children***  Mixed ethnicities: _t_ 50%, _c_ 64%  Caucasian: _t_ 50%, _c_ 36%  Hispanic: _t_ 31%, _c_ 8% |
| 23 | 109 | USA | N_t_ 10 | 9-16 years | 39.1 (7.7) | not reported | 100% | Caucasian 60%  African American 40% |
| 24 | 118 | GER | N_(all)_375 | 5.3 (-) | _t_ 35.8 (7.7)  _c_ 36.2 (8.5) | 45% | 100% | not reported |
| 25 | 135 | USA | N_t_ 9 | 11.0 (1.9) | 39.9 (5.5) | 55.6% | 100% | ***Parent***  Latino 100% |
| 26 | 143 | Spain | N_t_ 15  N_c_ 16 | 10.6 (-) | 39 (-) | not reported | not reported | not reported |
| 27 | 146 | USA | N_t_ 98  N_c_ 69 | 11.2 (-) | not reported | not reported | not reported | ***Families***  African American 100% |
| 28 | 173  176 | USA | N_t_ 75 | 10.4 (2.4) | 35.3 (5.8) | 46% | 73.3% | ***Families***  White 77%,  African American 18%,  Other 5% |
| 29 | 164 | UK | N_t_ 10 | 7-14 years | not reported | not reported | not reported | not reported |
| 30 | 200 | USA | N_t_ 45  N_c_ 49 | _t_ 14.4 (1.4)  _c_ 14.7 (1.5) | _t_ 42.4 (5.4)  _c_40.6 (5.1) | _t_24%  _c_32% | _t_37%  _c_38% | ***Children***  Non-White: _t_8%, _c_2%  ***Parents***  Non-White: _t_4%, _c_1% |
| 31 | 92 | USA | N_t_ 25  N_c_ 18 | _t_ 12.8 (-)  _c_ 10.8 (-) | _t_ 39.8 (-)  _c_ 37.8 (-) | _t_56%  _c_27.8% | 100% | ***Children***  White: _t_76%, _c_ 50%  ***Parents***  White: _t_84%, _c_ 72.2% |
| 32 | 208 | USA | N_t_ 43  N_c_ 44 | _t_ 10.4 (3.3)  _c_ 10.9 (3.3) | _t_ 37.8 (9.1)  _c_ 37.3 (7.5) | _t_47.1%  _c_48.0% | _t_94.1%  _c_80.0% | ***Children***  Hispanic/Latinx _t_ 35.3%, _c_ 40.4%  ***Parents***  Hispanic/Latinx: _t_ 29.4%, _c_ 31.9% |
| 33 | 6  202 | USA | N_t_ 61 children  N_c_ 66 children | _t_13.2 (2.7)  _c_13.3 (2.5) | not reported | _t_60.7%  _c_68.2% | not reported | ***Children***  Non-White _t_19.7%, _c_ 15.2% |
| 34 | 205  211 | China | N_t_ 34  N_c_ 27 | 8-18 years | not reported | _t_39.2%  _c_48.0% | _t_88.2%  _c_81.5% | not reported |
| 35 | 212 | Iran | N_t_ 30  N_c_ 30 | 8-16 years | not reported | not reported | not reported | not reported |
| 36 | 94 | USA | NC_t_ 21  NC_c_ 19 | _t_ 12.2 (2.8)  _c_ 12.3 (2.9) | not reported | _t_47.6%  _c_36.8% | not reported | ***Children***  Non-White: _t_ 14.3%, _c_ 5.3% |
| 37 | 2 | Sweden | 8 parents  7 children | Range 8-17 | not reported | 57.1% | 75% | not reported |
| 38 | 15 | AUS | 15 parents  8 children  6 siblings | 13.1  Range 9-17 | 51.5  Range 43-65 | 58.3% | not reported | ***Families***  Caucasian 95.3%  Indian 4.7% |
| 40 | 59  214 | UK | 5 parents  6 children  101 parents  138 children | 4-16 years | not reported | not reported | not reported | not reported |
| 41 | 79 | UK | 36 parents  37 children | 8-17 years | not reported | 52.4% | 74.7% | ***Families***  White British 68.8% |
| 43 | 206 | IRE | 23 parents  7 partners  15 children | 5-18 years | not reported | not reported | 78.2% | not reported |
| 44 | 213 | AUS | 10 parents | 8.5 (1.4) | 39.5 (2.9) | 50% | 100% | not reported |

## Overview of papers included in review with assigned IDs for trial (ID-t) and paper (ID-n)

Table 2S Study IDs, record IDs and references

| Study (ID-t) | Intervention | Report (ID-n) | References |
| --- | --- | --- | --- |
| 1 | Family Talk Intervention | 1 | Giannakopoulos, G. and Solantaus, T. and Tzavara, C. and Kolaitis, G. (2021). Mental health promotion and prevention interventions in families with parental depression: A randomized controlled trial |
| 2 | Family Talk Intervention | 73 | Christiansen, H. and Anding, J. and Schrott, B. and Rohrle, B. (2015). Children of mentally ill parents-a pilot study of a group intervention program |
| 3 | Family Talk Intervention | 93 | Punamaki, R. L. and Paavonen, J. and Toikka, S. and Solantaus, T. (2013). Effectiveness of preventive family intervention in improving cognitive attributions among children of depressed parents: a randomized study |
|  |  | 119 | Solantaus, Tytti and Toikka, Sini and Alasuutari, Maarit and Beardslee, William R. and Paavonen, E. (2010)Preventive interventions in families with parental depression: children's psychosocial symptoms and prosocial behaviour |
|  |  | 123 | Solantaus, Tytti and Toikka, Sini and Alasuutari, Maarit and Beardslee, William R. and Paavonen, E. (2009) Safety, feasibility and family experiences of preventive interventions for children and families with parental depression |
| 4 | Beardslee Family Intervention | 186 | Beardslee, W. R. and Hoke, L. and Wheelock, I. and Rothberg, P. C. and van de Velde, P. and Swatling, S. (1992). Initial findings on preventive intervention for families with parental affective disorders |
| 5 | Beardslee Family Intervention | 180 | Beardslee, W. R. and Wright, E. and Rothberg, P. C. and Salt, P. and Versage, E. (1996) Response of families to two preventive intervention strategies. Long-term differences |
| 6 | Beardslee Family Intervention | 148 | Beardslee, W. R., Wright, E. J., Gladstone, T. R., & Forbes, P. (2007). Long-term effects from a randomized trial of two public health preventive interventions for parental depression. |
|  |  | 177 | Beardslee, W. R., Wright, E. J., Salt, P., Drezner, K., Gladstone, T. R., Versage, E. M., & Rothberg, P. C. (1997). Examination of children's responses to two preventive intervention strategies over time. |
|  |  | 178 | Beardslee, W. R., Salt, P., Versage, E. M., Gladstone, T. R., Wright, E. J., & Rothberg, P. C. (1997). Sustained change in parents receiving preventive interventions for families with depression |
|  |  | 179 | Beardslee, W. R., Versage, E. M., Wright, E. J., Salt, P., Rothberg, P. C., Drezner, K., & Gladstone, T. R. G. (1997). Examination of preventive interventions for families with depression: Evidence of change |
|  |  | 162 | Beardslee, W. R., Gladstone, T. R., Wright, E. J., & Cooper, A. B. (2003). A family-based approach to the prevention of depressive symptoms in children at risk: evidence of parental and child change |
| 7 | Mobile-enhanced family-focused  therapy (M-FFT) | 5 | Miklowitz, D. J. and Weintraub, M. J. and Posta, F. and Walshaw, P. D. and Frey, S. J. and Morgan-Fleming, G. M. and Wilkerson, C. A. and Denenny, D. M. and Arevian, A. A. (2020).Development and Open Trial of a Technology-Enhanced Family Intervention for Adolescents at Risk for Mood Disorders |
| 8 | Coping and Promoting Strength (CAPS) | 8 | Ginsburg, G. S., Tein, J. Y., & Riddle, M. A. (2021). Preventing the onset of anxiety disorders in offspring of anxious parents: A six-year follow-up. |
|  |  | 20 | Schleider, J. L., Ginsburg, G. S., & Drake, K. (2018). Perceived peer victimization predicts anxiety outcomes in a prevention program for offspring of anxious parents |
|  |  | 34 | Pella, J. E., Drake, K. L., Tein, J. Y., & Ginsburg, G. S. (2017). Child anxiety prevention study: Impact on functional outcomes. Child Psychiatry & Human Development, 48, 400-410. |
|  |  | 70 | Ginsburg, G. S., Drake, K. L., Tein, J. Y., Teetsel, R., & Riddle, M. A. (2015). Preventing onset of anxiety disorders in offspring of anxious parents: a randomized controlled trial of a family-based intervention. |
| 9 | Coping and Promoting Strength (CAPS) | 132 | Ginsburg, G. S (2009) The Child Anxiety Prevention Study: intervention model and primary outcomes |
| 10 | Young Smiles | 10 | Abel, K. M. and Bee, P. and Gega, L. and Gellatly, J. and Kolade, A. and Hunter, D. and Callender, C. et al. (2020) An intervention to improve the quality of life in children of parents with serious mental illness: the Young SMILES feasibility RCT |
| 11 | Ecologically based family therapy (EBFT) | 11 | Wu, Q., & Slesnick, N. (2019). Interruption of dysfunctional mother–child reciprocal influences associated with family therapy |
|  |  | 19 | Zhang, J., Slesnick, N., & Feng, X. (2018). Co‐Occurring Trajectory of Mothers’ Substance Use and Psychological Control and Children's Behavior Problems: The Effects of a Family Systems Intervention |
|  |  | 31 | Bartle‐Haring, S., Slesnick, N., & Murnan, A. (2018). Benefits to children who participate in family therapy with their substance‐using mother. |
|  |  | 48 | Slesnick, N., & Zhang, J. (2016). Family systems therapy for substance-using mothers and their 8-to 16-year-old children. |
| 12 | Ecologically based family therapy (EBFT) | 21 | Murnan, Aaron and Wu, Qiong and Slesnick, Natasha (2018). Effects of ecologically-based family therapy with substance-using, prostituting mothers. |
| 13 | GuG auf | 201 | Johanna Löchner, Kornelija Starman‑Wöhrle, Keisuke Takano, Lina Engelmann, Alessandra Voggt, Fabian Loy, Mirjam Bley, Dana Winogradow, Stephanie Hämmerle, Esther Neumeier, Inga Wermuth, Katharina Schmitt, Frans Oort, Gerd Schulte‑Körne and Belinda Platt. (2019) A randomised controlled trial of a family‑group cognitive‑behavioural (FGCB) preventive intervention for the children of parents with depression: short‑term effects on symptoms and possible mechanism |
|  |  | 16 | QUAL see below |
| 14 | Family Group Cognitive-Behavioral | 18 | Breslend, N. L., Parent, J., Forehand, R., Peisch, V., & Compas, B. E. (2019). Children of parents with a history of depression: The impact of a preventive intervention on youth social problems through reductions in internalizing problems. |
|  |  | 29 | Bettis, A. H., Forehand, R., Sterba, S. K., Preacher, K. J., & Compas, B. E. (2018). Anxiety and depression in children of depressed parents: Dynamics of change in a preventive intervention. |
|  |  | 72 | Compas, B. E., Forehand, R., Thigpen, J., Hardcastle, E., Garai, E., McKee, L., ... & Sterba, S. (2015). Efficacy and moderators of a family group cognitive–behavioral preventive intervention for children of parents with depression |
|  |  | 82 | McKee, L. G., Parent, J., Forehand, R., Rakow, A., Watson, K. H., Dunbar, J. P., ... & Compas, B. E. (2014). Reducing youth internalizing symptoms: Effects of a family-based preventive intervention on parental guilt induction and youth cognitive style |
|  |  | 138 | Compas, B. E., Forehand, R., Keller, G., Champion, J. E., Rakow, A., Reeslund, K. L., ... & Cole, D. A. (2009). Randomized controlled trial of a family cognitive-behavioral preventive intervention for children of depressed parents. |
|  |  | 116 | Compas, B. E., Forehand, R., Thigpen, J. C., Keller, G., Hardcastle, E. J., Cole, D. A., ... & Roberts, L. (2011). Family group cognitive–behavioral preventive intervention for families of depressed parents: 18-and 24-month outcomes. |
|  |  | 105 | Forehand, R., Thigpen, J. C., Parent, J., Hardcastle, E. J., Bettis, A., & Compas, B. E. (2012). The role of parent depressive symptoms in positive and negative parenting in a preventive intervention |
|  |  | 122 | Compas, B. E., Champion, J. E., Forehand, R., Cole, D. A., Reeslund, K. L., Fear, J., ... & Roberts, L. (2010). Coping and parenting: Mediators of 12-month outcomes of a family group cognitive–behavioral preventive intervention with families of depressed parents. |
| 15 | Kanu | 26 | Fernando, Silvia Carvalho and Griepenstroh, Julia and Bauer, Ullrich and Beblo, Thomas and Driessen, Martin (2018). Primary prevention of mental health risks in children of depressed patients: Preliminary results from the Kanu-intervention |
| 16 | The Renascent Children's Program | 45 | Usher, Amelia M. and McShane, Kelly E. (2016). Supporting children of substance abusing families: Preliminary outcomes of the renascent children's program |
| 17 | Parent- AdolescenCBT | 62 | Spirito, A. and Wolff, J. C. and Seaboyer, L. M. and Hunt, J. and Esposito-Smythers, C. and Nugent, N. and Zlotnick, C. and Miller, I. (2015) Concurrent Treatment for Adolescent and Parent Depressed Mood and Suicidality: Feasibility, Acceptability, and Preliminary Findings |
| 18 | Supporting Kids and Their Environment (SKATE) | 67 | Lewis, Andrew J. and Holmes, Natalie-Mai and Watkins, Brittany and Mathers, Donna (2015). Children impacted by parental substance abuse: An evaluation of the Supporting Kids and Their Environment program |
| 19 | CBT with mother-child interaction (CBT-MCI) | 71 | Creswell, C. and Cruddace, S. and Gerry, S. and Gitau, R. and McIntosh, E. and Mollison, J. et al. (2015). Treatment of childhood anxiety disorder in the context of maternal anxiety disorder: a randomised controlled trial and economic analysis. |
| 21 | Fortalezas Familiares (Family Strengths) | 91 | Valdez, C. R. and Padilla, B. and Moore, S. M. and Magana, S. (2013). Feasibility, acceptability, and preliminary outcomes of the Fortalezas Familiares intervention for latino families facing maternal depression |
| 22 | Project Hope | 101 | Mason, W. A. and Haggerty, K. P. and Fleming, A. P. and Casey-Goldstein, M. (2012). Family Intervention to Prevent Depression and Substance Use Among Adolescents of Depressed Parents |
| 23 | Keeping Families Stronger Intervention (KFS) | 109 | Valdez, C. R. and Mills, C. L. and Barrueco, S. and Leis, J. and Riley, A. W. (2011). A Pilot Study of a Family-Focused Intervention for Children and Families Affected by Maternal Depression |
| 24 | EFFEKT-E | 118 | Buhler, A. and Kotter, C. and Jaursch, S. and Losel, F. (2011). Prevention of familial transmission of depression: EFFEKT-E, a selective program for emotionally burdened families |
| 25 | PIP (Prevention Intervention Program) | 135 | D'Angelo, E. J. and Llerena-Ouinn, R. and Shapiro, R. and Colon, F. and Rodriguez, P. and Gallagher, K. and Beardslee, W. R. (2009). Adaptation of the preventive intervention program for depression for use with predominantly low-income Latino families |
| 26 | Family Competence Programme (FCP), adaptation of Strengthening Families Programme | 143 | Orte, Carmen and Touza, Carmen and Ballester, Lluis and March, Marti (2008). Children of drug-dependent parents: Prevention programme outcomes. *Educational Research*, *50*(3), 249-260 |
| 27 | Strong African American Families (SAAF) programme | 146 | Beach, Steven R. and Kogan, Steven M. and Brody, Gene H. and Chen, Yi-Fu and Lei, Man-Kit and Murry, Velma M. (2008). Change in caregiver depression as a function of the Strong African American Families Program |
| 28 | Focus on Families | 176 | Catalano, R. F., Haggerty, K. P., Gainey, R. R., & Hoppe, M. J. (1997). Reducing parental risk factors for children's substance misuse: Preliminary outcomes with opiate-addicted parent |
|  |  | 173 | Catalano, R. F., Gainey, R. R., Fleming, C. B., Haggerty, K. P., & Johnson, N. O. (1999). An experimental intervention with families of substance abusers: one‐year follow‐up of the focus on families project. |
| 29 | Strength to Strength | 164 | Place, M. and Reynolds, J. and Cousins, A. and O'Neill, S. (2002). Developing a Resilience Package for Vulnerable Children |
| 30 | Adolescent coping with Stress Course | 200 | Clarke, G. N., Hornbrook, M., Lynch, F., Polen, M., Gale, J., Beardslee, W., ... & Seeley, J. (2001). A randomized trial of a group cognitive intervention for preventing depression in adolescent offspring of depressed parents |
| 31 | Multisystemic Therapy-Building Stronger Families | 92 | Schaeffer, C. M. and Swenson, C. C. and Tuerk, E. H. and Henggeler, S. W (2013). Comprehensive treatment for co-occurring child maltreatment and parental substance abuse: outcomes from a 24-month pilot study of the MST-Building Stronger Families program |
| 32 | Multisystemic Therapy-Building Stronger Families | 208 | Schaeffer, C. M., Swenson, C. C., & Powell, J. S. (2021). Multisystemic Therapy-Building Stronger Families (MST-BSF): Substance misuse, child neglect, and parenting outcomes from an 18-month randomized effectiveness trial |
| 33 | Family-focused therapy | 6 | Miklowitz, D. J., Schneck, C. D., Walshaw, P. D., Singh, M. K., Sullivan, A. E., Suddath, R. L., ... & Chang, K. D. (2020). Effects of family-focused therapy vs enhanced usual care for symptomatic youths at high risk for bipolar disorder: a randomized clinical trial |
|  |  | 202 | Miklowitz, D. J., Merranko, J. A., Weintraub, M. J.,, Walshaw, P. D., Singh, M. K., Chang, K. D., & Schneck, C. D. (2020). Effects of family-focused therapy on suicidal ideation and beahvior in youth at high risk for bipolar disorder |
| 34 | Multiple family therapy (MFT) | 205 | Ma, J. L. C.; Xia, L. L. L.; Yau-Ng, M.; Yiu, Y. C. (2022). Treatment efficacy of multiple family therapy in helping Chinese children of depressed parents in Hong Kong, China |
|  |  | 211 | Ma, Xia, L. L. L., Yau-Ng, M., & Yan-Yee, C. (2023). Treatment Efficacy of Multiple Family Therapy in Helping Hong Kong Chinese Parents Recover from Depression |
| 35 | Family Friendly Program (FFP) | 212 | Rahmani, N.; Teymuri, S.; Bayazi, Mohammad Hossein; Rajaei, Ali Reza (2022) Investigating the effect of implementing a family-friendly program (based on the mbct approach with parent-child interaction) on the reduction of parental anxiety sensitivity and separation anxiety disorder in children and adolescents |
| 36 | Family-focused therapy | 94 | Miklowitz, D. J., Schneck, C. D., Singh, M. K., Taylor, D. O., George, E. L., Cosgrove, V. E., Howe, M. E., Dickinson, L. M., Garber, J., & Chang, K. D. (2013). Early intervention for symptomatic youth at risk for bipolar disorder: a randomized trial of family-focused therapy |
| Qualitative studies | | |  |
| 37 | Beardslee Family Intervention | 2 | Strand, Jennifer and Meyersson, Niklas (2020) Parents with psychosis and their children: experiences of Beardslee's intervention. |
| 10 | Young Smiles | 10 | Abel, K. M. and Bee, P. and Gega, L. and Gellatly, J. and Kolade, A. and Hunter, D. and Callender, C. and Carter, L. A. et al. (2020). An intervention to improve the quality of life in children of parents with serious mental illness: the Young SMILES feasibility RCT |
| 38 | The Family model | 15 | Hoadley, Benjamin and Falkov, Adrian and Agalawatta, Neelya (2019).The acceptability of a single session family focused approach for children/young people and their parents attending a child and youth mental health service |
| 13 | GuG auf | 16 | Claus, N. and Marzano, L. and Loechner, J. and Starman, K. and Voggt, A. and Loy, F. and Wermuth, I. and Haemmerle, S. and Engelmann, L. and Bley, M. and Schulte-Koerne, G. and Platt, B. (2019). Qualitative evaluation of a preventive intervention for the offspring of parents with a history of depression |
| 40 | KidsTime | 59 | Wolpert, M. and Hoffman, J. and Martin, A. and Fagin, L. and Cooklin, A (2015). An exploration of the experience of attending the Kidstime programme for children with parents with enduring mental health issues: Parents' and young people's views |
|  |  | 214* | Martin, A , Hoffman J, Nolas, S.M., Kamenopoulou, L., Wolper, M. (2011). Evaluation Report: For the Evaluation of the KidsTime Workshops (2010-2011). *only some quantitative data from feedback forms for family functioning |
| 41 | Moving Parents and Children Together Programme (M-PACT) | 79 | Templeton, Lorna (2014). Supporting families living with parental substance misuse: the M-PACT (Moving Parents and Children Together) programme |
| 4 | Beardslee Family Intervention | 186 | Beardslee, W. R. and Hoke, L. and Wheelock, I. and Rothberg, P. C. and van de Velde, P. and Swatling, S.(1992) Initial findings on preventive intervention for families with parental affective disorders |
| 43 | Family Talk Intervention | 206 | Mulligan, C.; Furlong, M.; McGarr, S.; O'Connor, S.; McGilloway, S (2021) The Family Talk Programme in Ireland: A Qualitative Analysis of the Experiences of Families With Parental Mental Illness |
| 44 | CBT for parents and children | 213 | Galea, Samantha; Wade, Catherine; Salvaris, Chloe A.; Yap, Marie B. H.; Lawrence, Katherine A. (2022). Acceptability of an enhanced transdiagnostic CBT intervention for adults with anxiety disorders who are parenting an anxious child |

## Search Strategy

Excerpt of search strategy and terms used for Medline. Please see OSF project for full search terms per database: <https://osf.io/9uxgp/>

## Intervention components

Excerpt of coding of intervention components. Please see provided excel file (Intervention components table) or OSF project for full details: <https://osf.io/9uxgp/>

|  | Structural components | | | | | Components from psychotherapeutic approaches | | | | | Skills training | | | | | Psychoeducation (PE) | | | Building resources | | | |
| --- | --- | --- | --- | --- | --- | --- | --- | --- | --- | --- | --- | --- | --- | --- | --- | --- | --- | --- | --- | --- | --- | --- |
| Intervention | Assessment | Practical support | Goal setting | Facilitated parent-child interactions | Practice between sessions | Play/creativity/drama therapy | Systemic family therapy | Multi-family or group therapy | Cognitive-behavioural therapy | Video feedback | Mindfulness/relaxation | Communication skills | Problem-solving and coping skills | Communication about PMI | Parenting | PE mental illness | PE PMI | PE child development | Family care plan | Building support networks children | Building support networks parents | Links other services /signposting |
| Coping and Promoting Strength (CAPS) |  |  |  |  |  |  |  |  | x |  | x | x | x | x | x | x |  |  |  |  |  |  |
| CBT mother-child interaction  (CBT-MCI) |  |  |  |  |  |  |  |  | x | x |  |  | x |  | x | x |  |  |  |  |  |  |
| Adolescent coping with Stress Course – adapted (ASC) | x |  |  |  | x |  |  | x | x |  |  |  | x |  |  |  |  |  |  |  |  |  |
| Ecologically based family therapy (EBFT) | x |  |  |  |  |  | x |  | x |  |  | x | x |  |  | x | x |  |  |  |  | x |
| Eltern- und Kinder-Training in emotional belasteten Familien (EFFEKT-E) |  |  |  |  | x | x |  |  | x |  |  |  | x |  | x |  |  | x |  | x | x |  |

## Definition of coded intervention components

**Assessment:** Assessment of family or individual strengths and weaknesses, or problems, at the start.

**Practical support:** Any practical support, e.g. helping to call someone.

**Setting goals for the intervention:** If family or individual goals for the treatment were set at the start.

**Facilitated parent-child interactions:** We do NOT code this component if they just talk about improving/stimulating/supporting the parent-child relationship or family cohesion. We DO code it, if they specify specific interactions together, e.g., playing during sessions (therapeutic play sessions as well as games) aimed at improving the relationship, learning about spending positive time with your child, ‘homework’ to spend time with child. We have NOT coded it if they merely have time together without mentioning this as an aim to improve relationship (eg. simply having sessions with parents and child involved).

**Encouraging practice in between sessions:** Usually referred to as homework. Also coded if the intervention description mentioned that participants had to practice between sessions.

**Play/creativity/drama therapy:** Any play, creativity, arts or games for children. This can be with a specific therapeutic goal, but equally for fun.

**Systemic family therapy:** Coded if the intervention referred to systemic theory, the ecological model or being systemically informed.

**Multi-family or group therapy:** Coded if the intervention used a group setting.

**Cognitive behavioural therapy:** This is coded if the intervention mentions to be based in CBT or using CBT or cognitive restructuring skills or strategies.

**Video feedback:** When video feedback to support parents in learning skills is being used.

**Mindfulness/relaxation:** This component reflects mentioning using mindfulness or relaxation techniques or elements in the intervention.

**Communication skills:** This is about learning communication *skills*. If communication an such is enhanced in the family, family meetings are organised, of families are supported to communicate about PMI, this was not coded here.

**Problem-solving and coping skills:** This reflects any problem-solving or coping skills for parents and/or children. This was initially coded for parents and children separately. However, most papers either taught those skills to all family members or didn't specify to whom. The only times when it was specified for one person, was if the intervention was targeted at the young person (for example) and parents were only informed or included in child treatment.

**Supporting families to communicate about PMI:** This reflects stimulating families to talk about PMI during and/or outside of sessions. This includes stimulating a family understanding of the PMI as this presumes having talked about it in the family.

**Parenting skills:** All parenting skills. coded if general parenting skills or specific skills are being mentioned. Also enhancement of parenting competence or confidence is scored. Psycho education about parenting is not coded (e.g. about cultural differences regarding parenting)

**Psychoeducation on mental illness:** This is about psychoeducation about mental health/illness in general, causes, symptoms etc

**Psychoeducation on impact of PMI:** This is about psychoeducation about the impact of PMI on families or the potential consequences of PMI for parenting and family life. This includes co-occurrence of MI in parent and child.

**Psychoeducation on child development:** This is about psychoeducation on child development in general.

**Developing a family care plan:** This was only coded if a clear and specific plan (e.g. safety or relapse plan) was developed to support the family after the intervention and which families could fall back upon in times of need. Examples included a relapse prevention plan or the detailing of post-intervention goals. Teaching families skills to keep their home safe was not scored, as this is not a plan they can use. Coping plans for children were also not coded as a family plan.

**Building support networks for children:** Activities that actively promote development of a support network. This does not include peer network through group session, but it does if there are ‘alumni’ sessions post-intervention. Also learning about friendship, identifying key people in network etc are coded.

**Building support networks for parents:** Same as for children but with focus on adults

**Links with other services and signposting:** coded if description talked about referring or signposting to other services. Also coded if the intervention was linked to other services or if information about other services was provided.

## Meta-analyses results

### Child internalising outcomes reported by child

Figures S1 to S8 show the forest plots for child reported internalising symptoms for the 4 different times of follow-up time points. Each meta-analysis was conducted with 2 different outcomes sets, as some studies used multiple measures to assess similar constructs.

Figure 1S is equal to Figure 1 in manuscript and is presented here for easier overview.


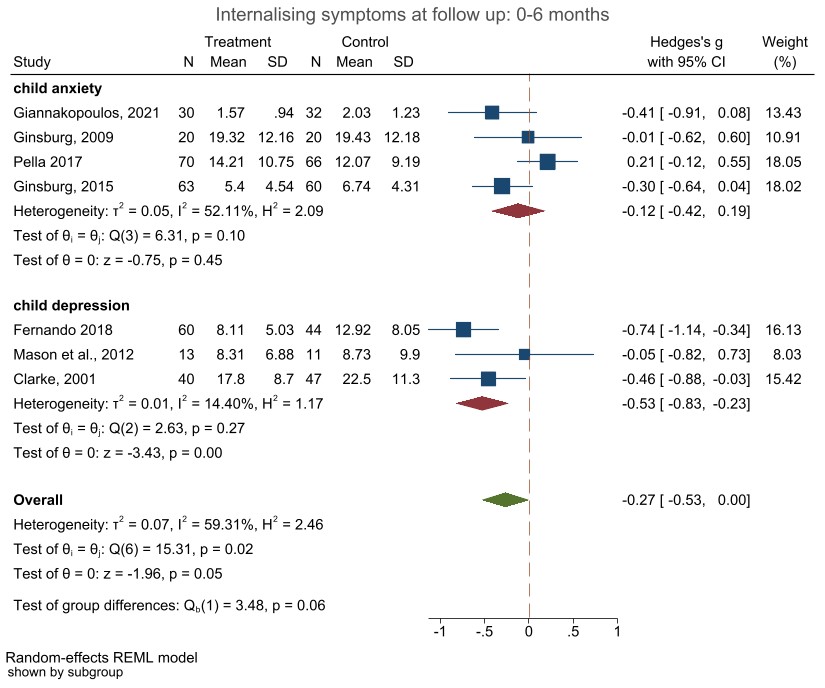


Figure 1S Forest plot child mental health outcomes (0-6 months) reported by child (a)


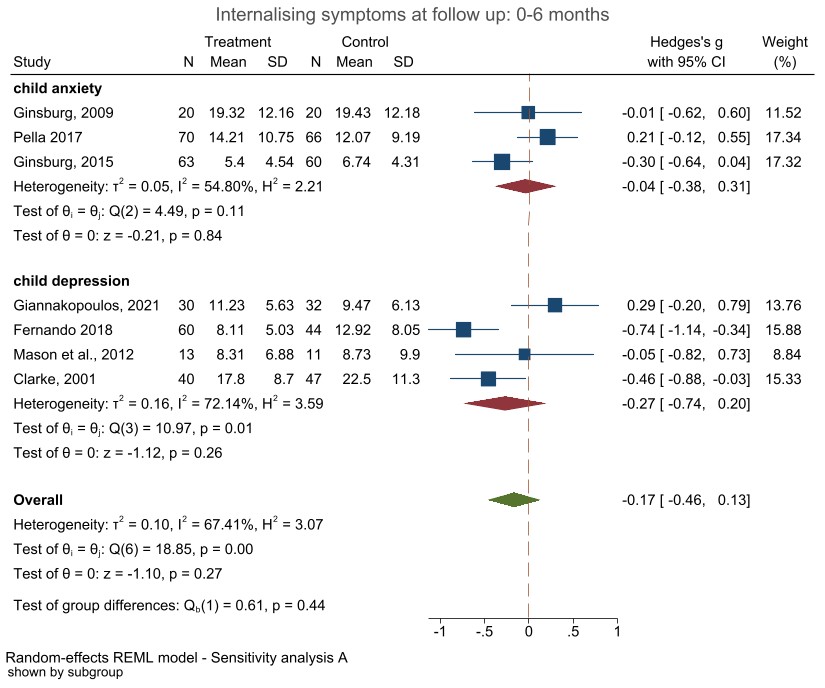


Figure 2S Forest plot child mental health outcomes (0-6 months) reported by child (b)


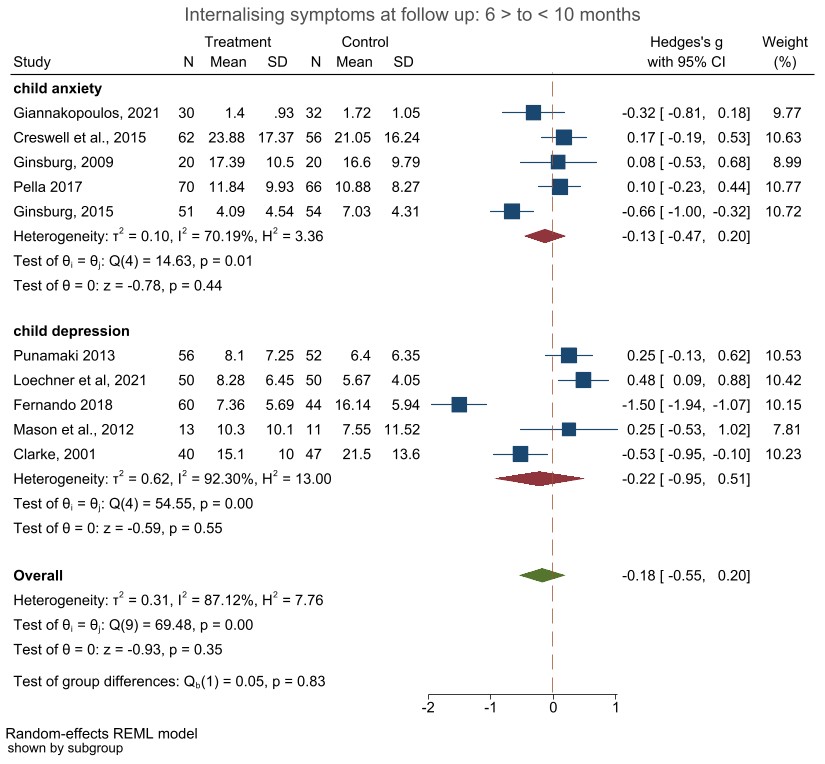


Figure 3S Forest plot child mental health outcomes (6-10 months) reported by child (a)


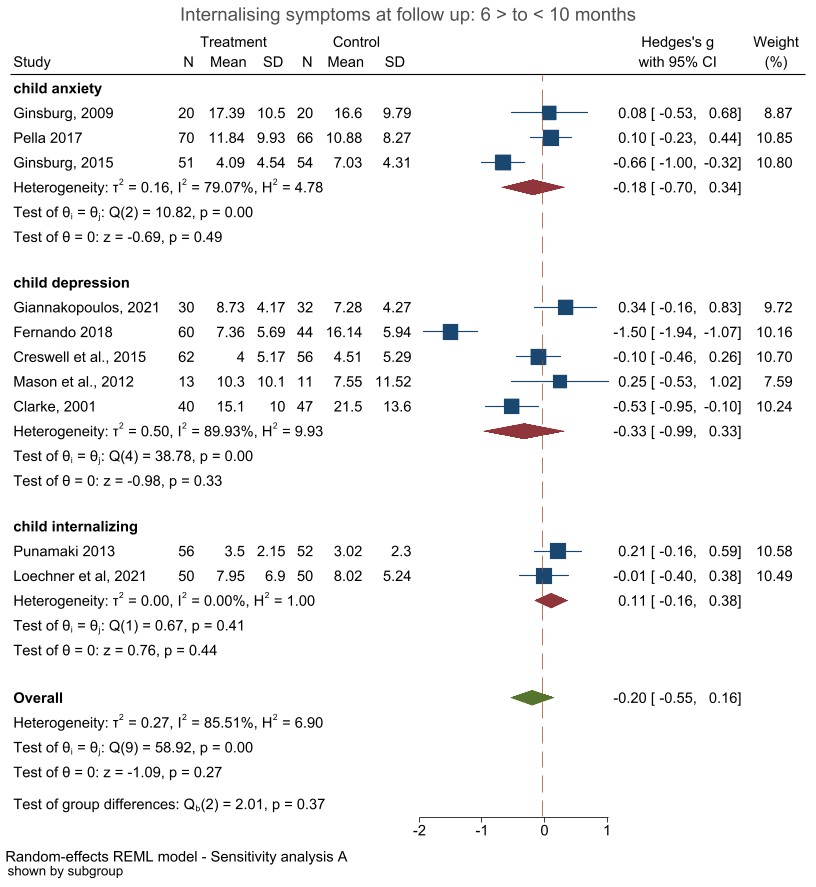


Figure 4S Forest plot child mental health outcomes (6-10 months) reported by child (b)


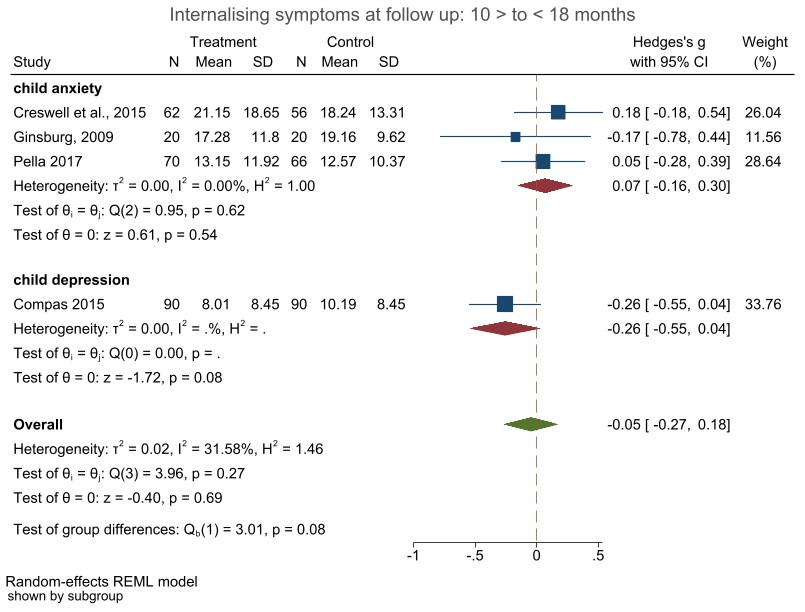


Figure 5S Forest plot child mental health outcomes (10-18 months) reported by child (a)


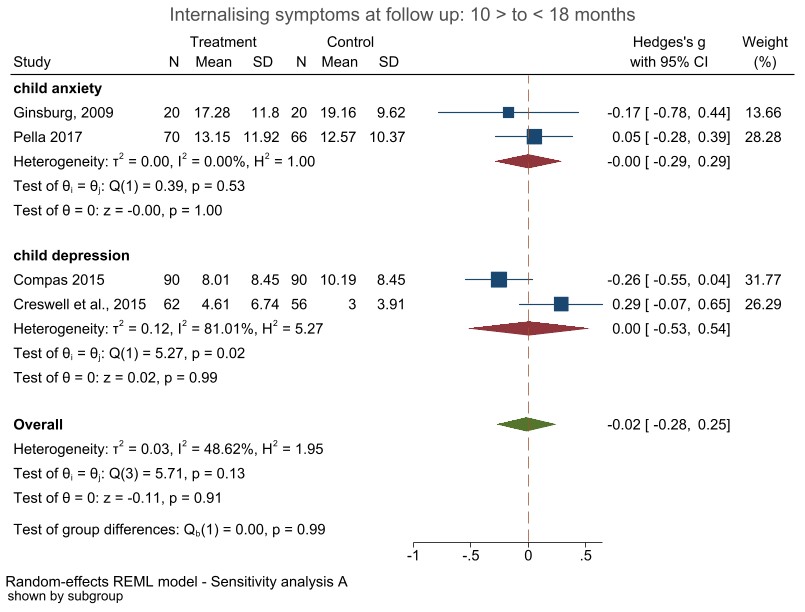


Figure 6S Forest plot child mental health outcomes (10-18 months) reported by child (b)


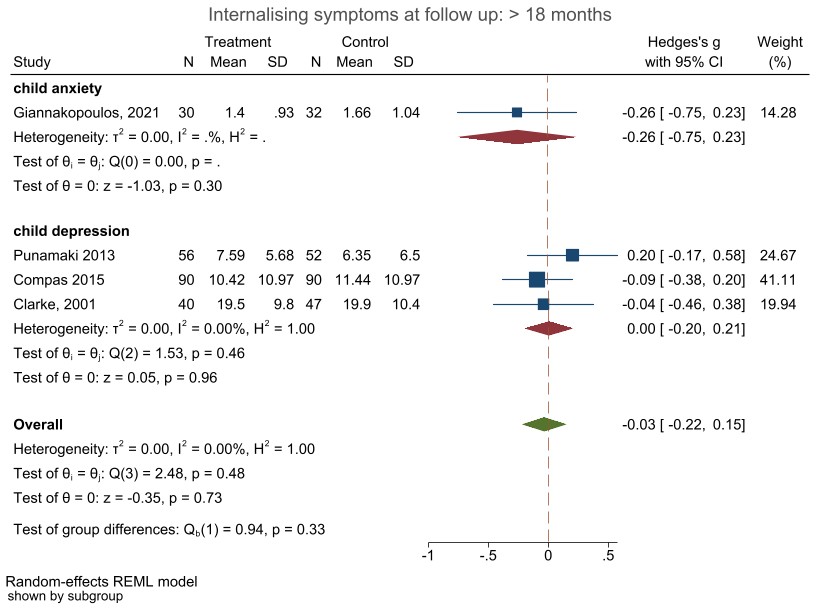


Figure 7S Forest plot child mental health outcomes (18+ months) reported by child (a)


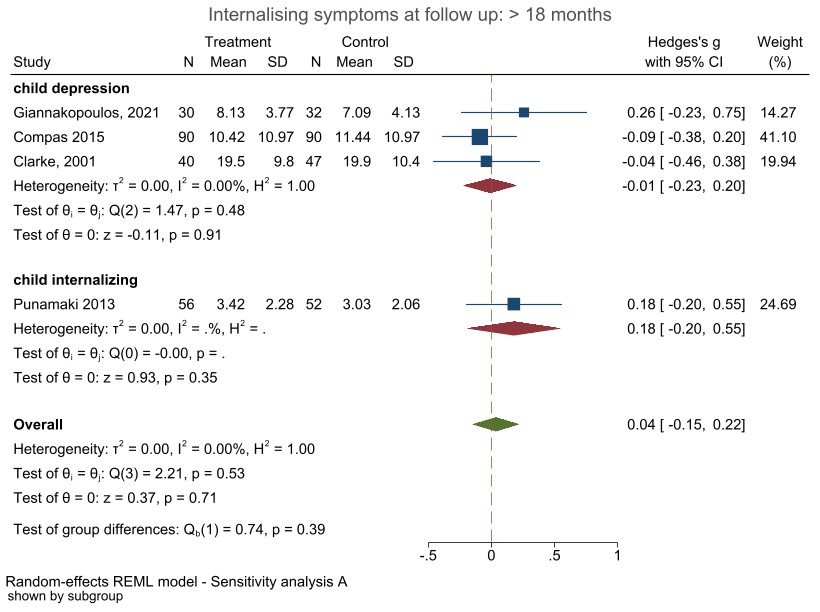


Figure 8S Forest plot child mental health outcomes (18+ months) reported by child (b)

### Child internalising outcomes reported by parents

Figures S9 to S16 show the forest plots for parent reported child internalising symptoms for the 4 different times of follow-up time points. Each meta-analysis was conducted with 2 different outcomes sets, as some studies used multiple measures to assess similar constructs. Figure 9S is equal to Figure 2 in manuscript and is presented here for easier overview.


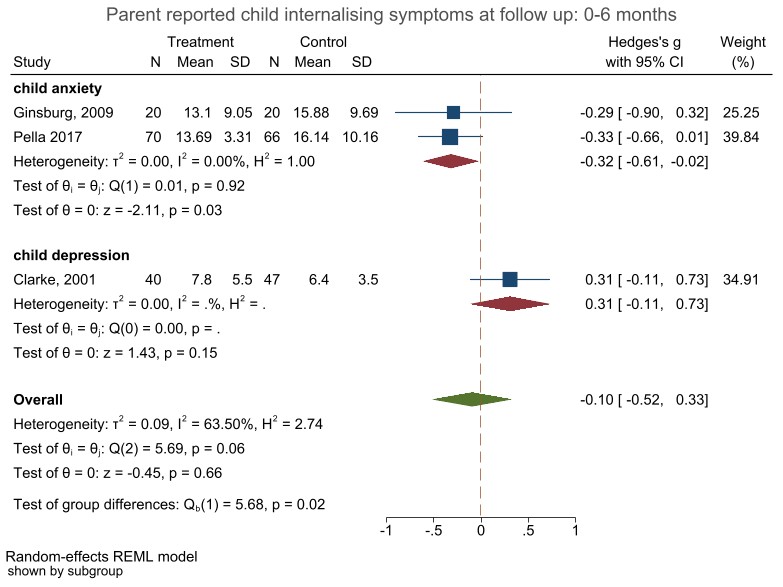


Figure 9S Forest plot child mental health outcomes (0-6 months) reported by parent (a)


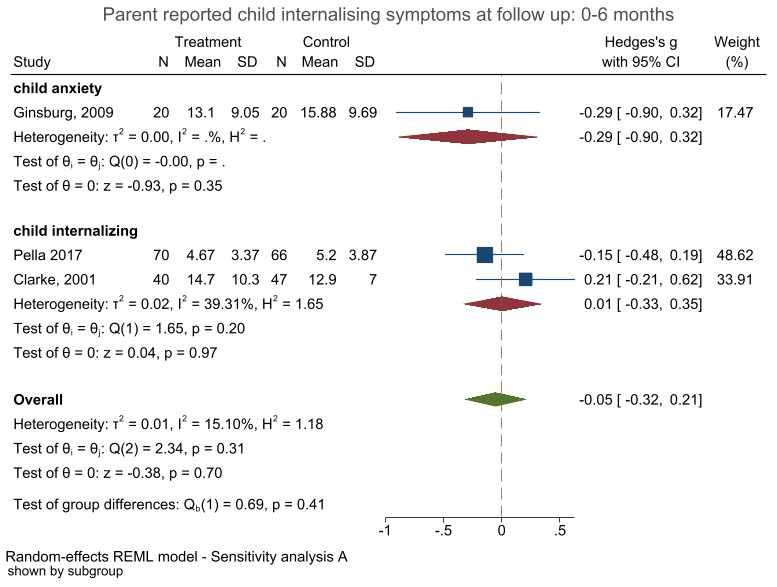


Figure 10S Forest plot child mental health outcomes (0-6 months) reported by parent (b)


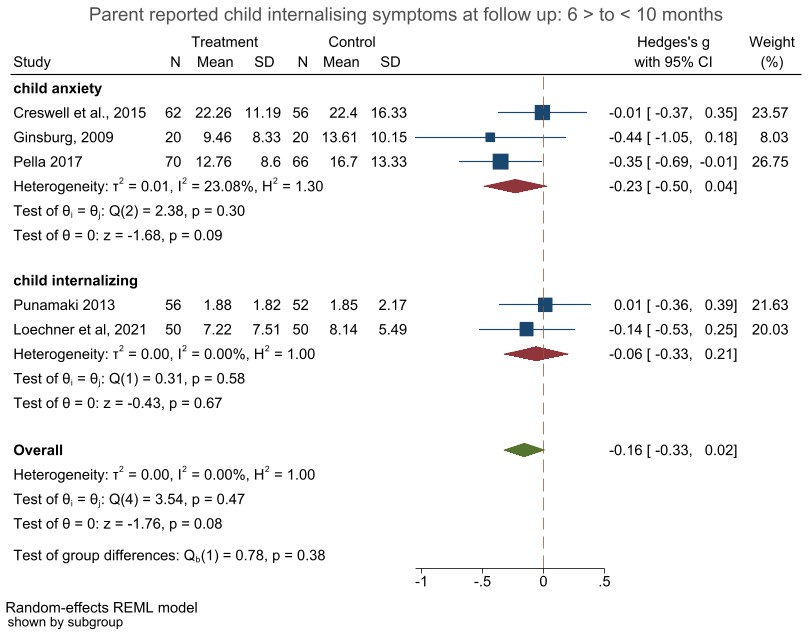


Figure 11S Forest plot child mental health outcomes (6-10 months) reported by parent (a)


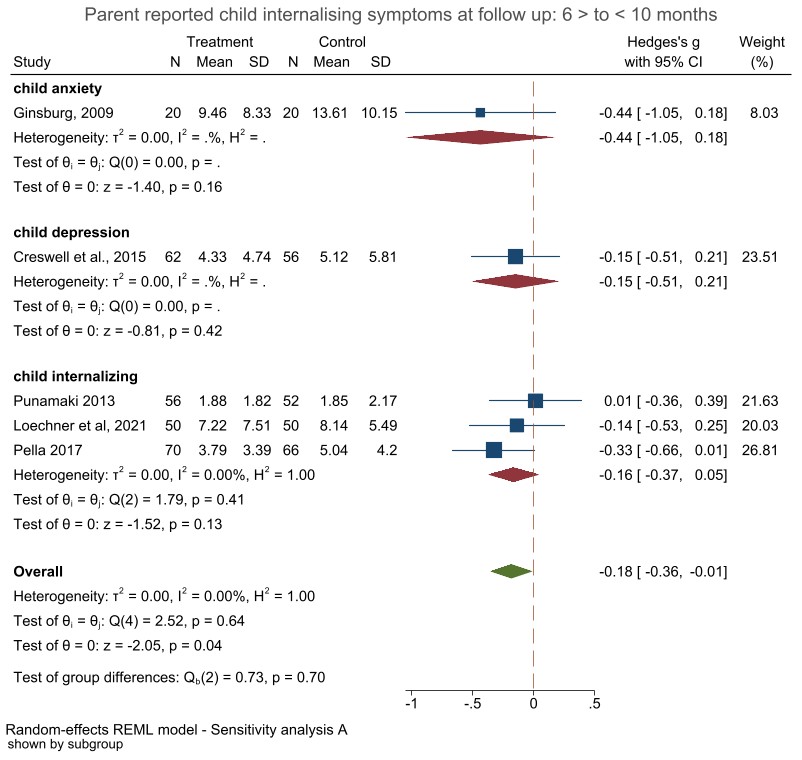


Figure 12S Forest plot child mental health outcomes (6-10 months) reported by parent (b)


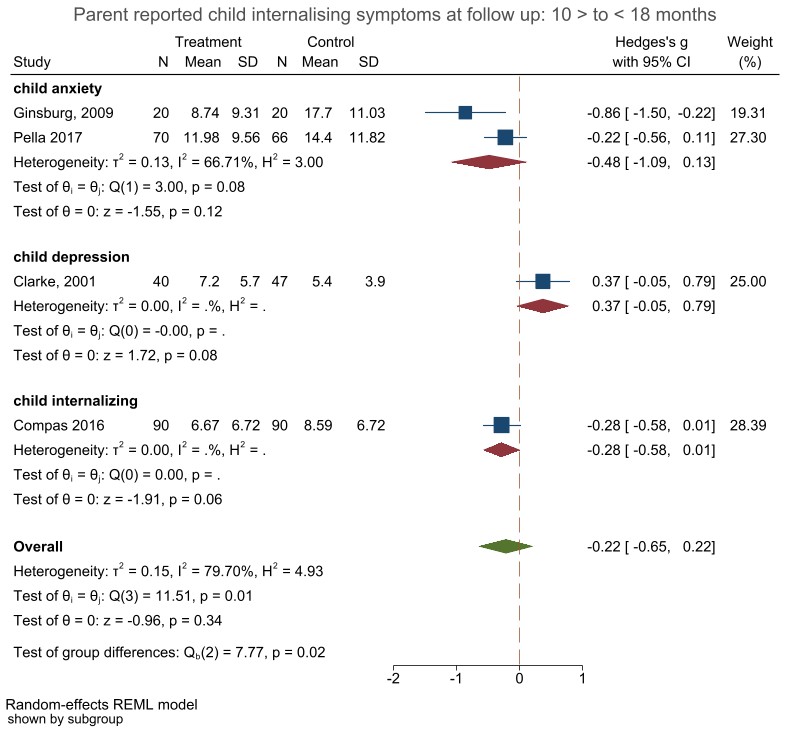


Figure 13S Forest plot child mental health outcomes (10-18 months) reported by parent (a)


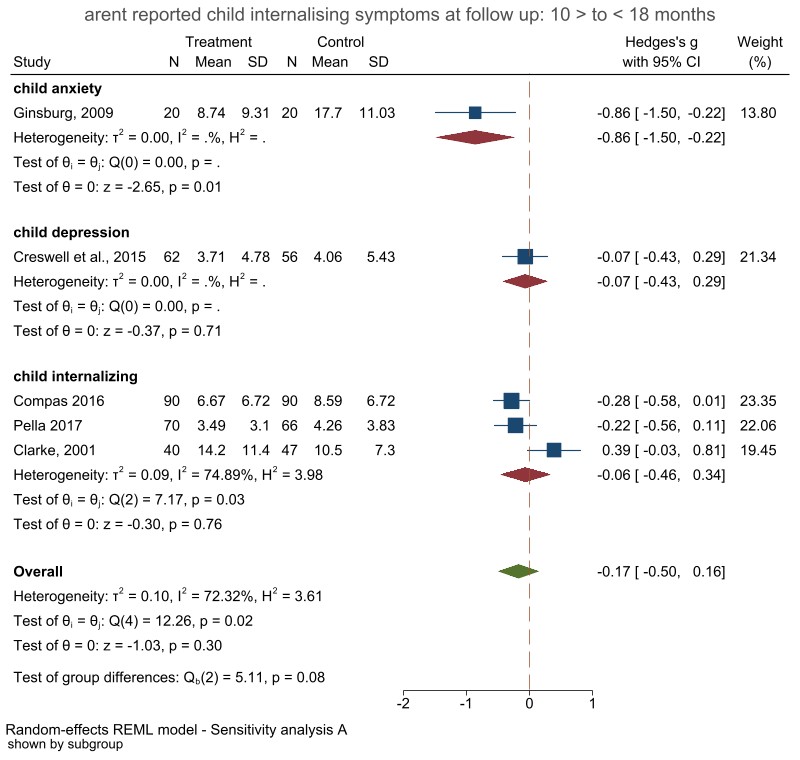


Figure 14S Forest plot child mental health outcomes (10-18 months) reported by parent (b)


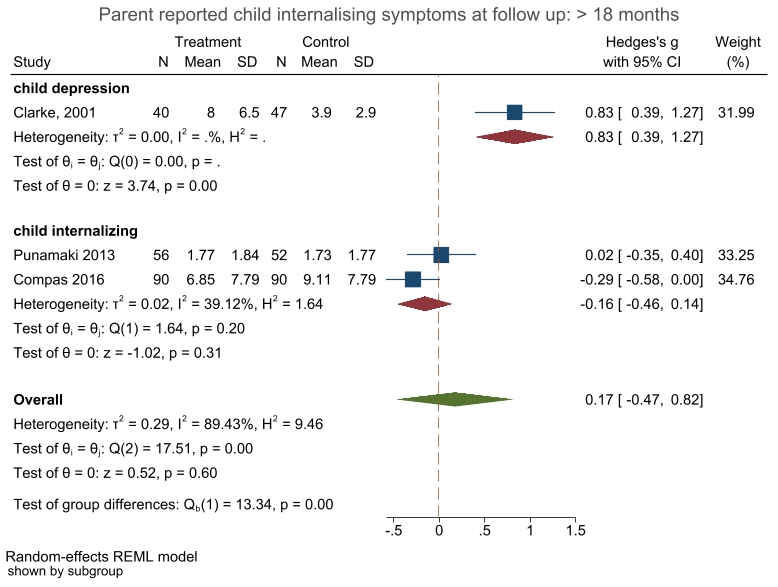


Figure 15S Forest plot child mental health outcomes (18+ months) reported by parent (a)


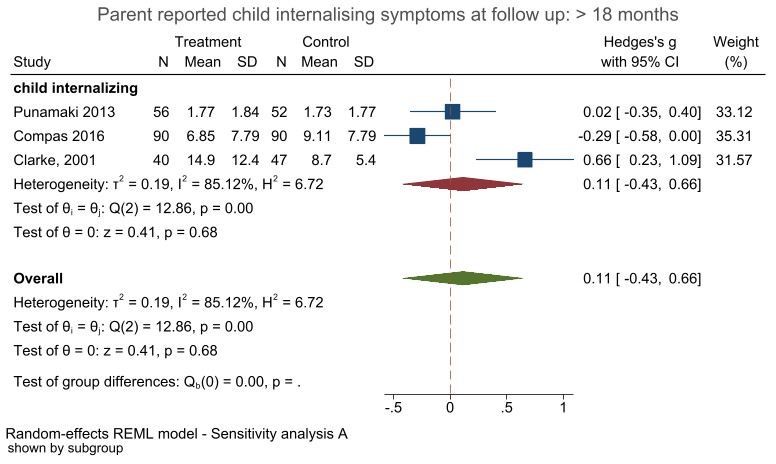


Figure 16S Forest plot child mental health outcomes (18+ months) reported by parent (b)

### Parent mental health outcomes

Figure 17S to 24S show forest plots for parent mental health outcomes for four follow-up time points. Each meta-analysis was conducted with 2 different outcomes sets, as some studies used multiple measures to assess similar constructs.


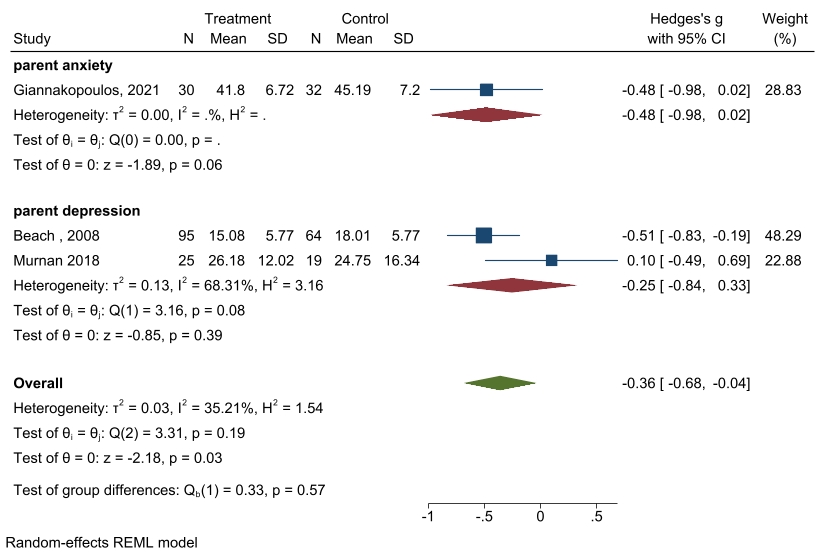


Figure 17S Forest plot for parent mental health outcomes at 0-6 months (a)


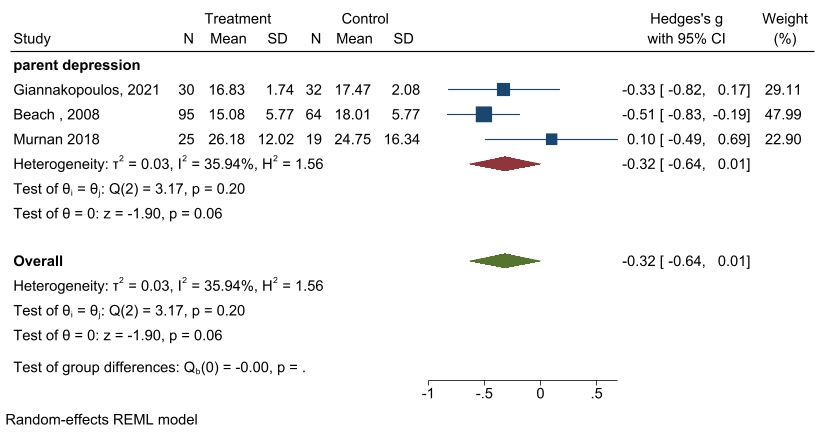


Figure 18S Forest plot for parent mental health outcomes at 0-6 months (b)


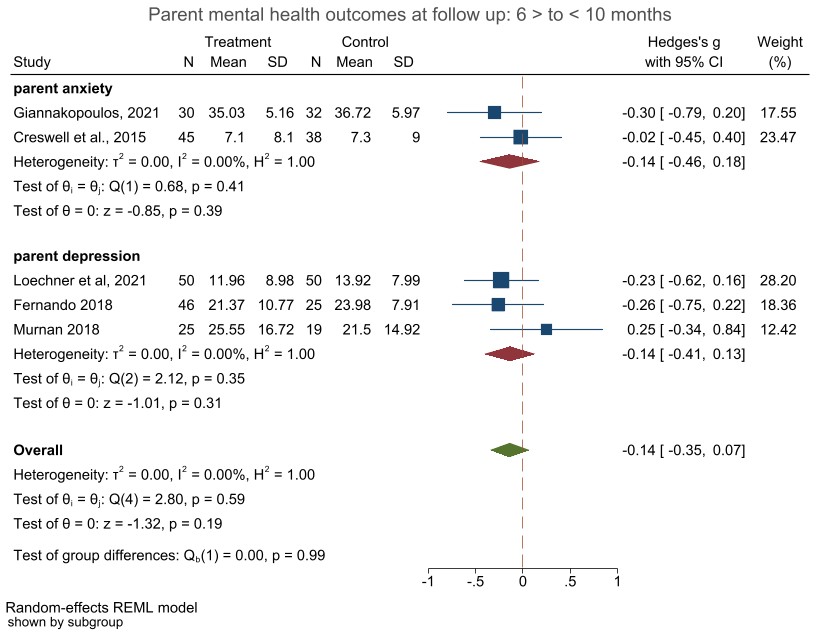


Figure 19S Forest plot for parent mental health outcomes at 6-10 months (a)


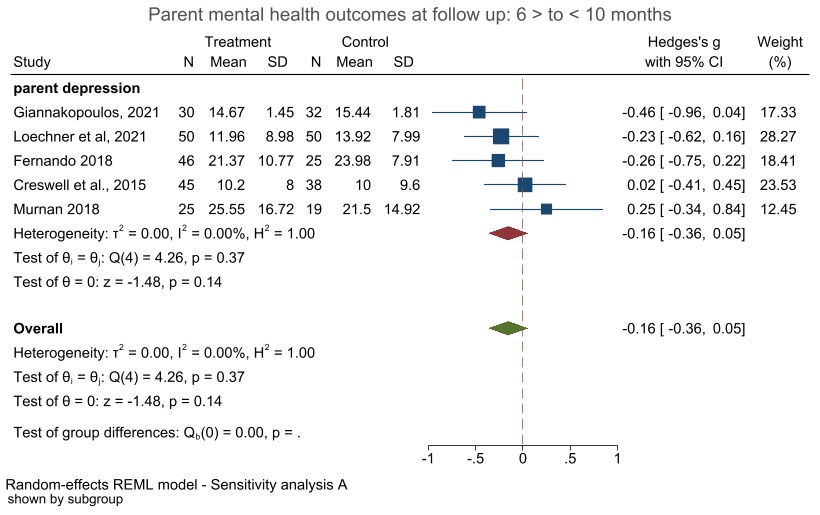


Figure 20S Forest plot for parent mental health outcomes at 6-10 months (b)


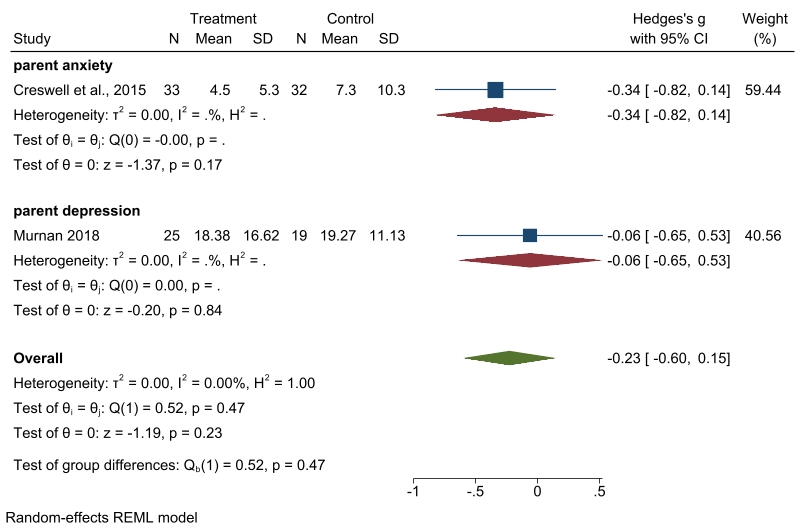


Figure 21S Forest plot for parent mental health outcomes at 10-18 months (a)


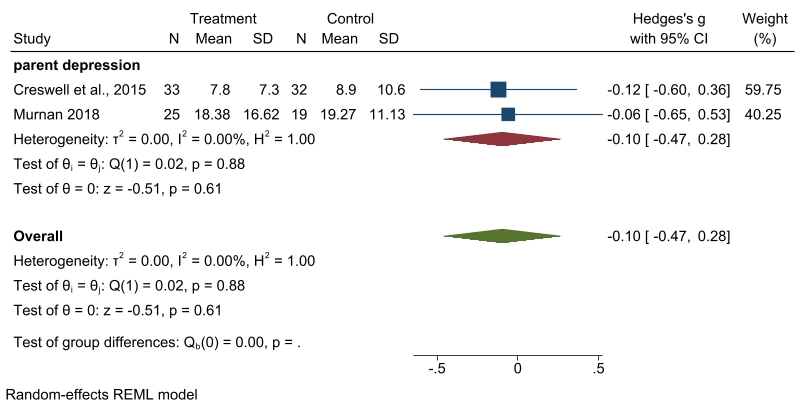


Figure 22S Forest plot for parent mental health outcomes at 10-18 months (b)


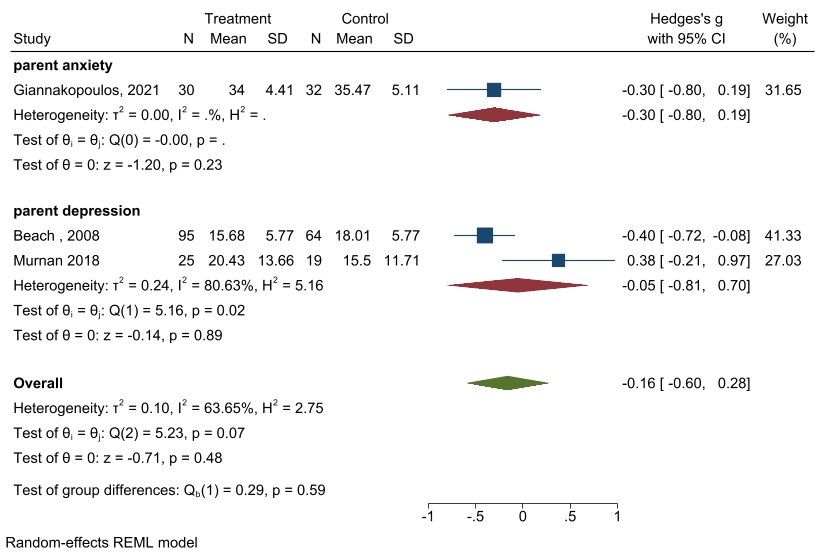


Figure 23S Forest plot for parent mental health outcomes at 18+ months (a)


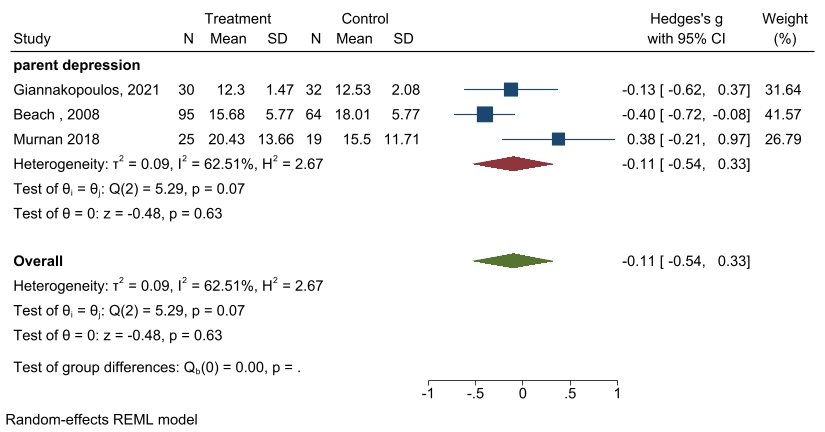


Figure 24S Forest plot for parent mental health outcomes at 18+ months (b)

## Meta-regressions

We only show results for meta-regressions that could be performed with sufficient number of studies.

### Meta-regression with child internalising outcomes reported by children at first follow-up

### Meta-regression with child internalising outcomes reported by children at second follow-up

### Meta-regression with child internalising outcomes reported by parents at first follow-up

### Meta-regression with child internalising outcomes reported by parents at second follow-up

### Meta-regression with parent outcomes reported at first follow-up

## Effect sizes across follow-up time

### Effect sizes of child internalising problems per study and follow-up


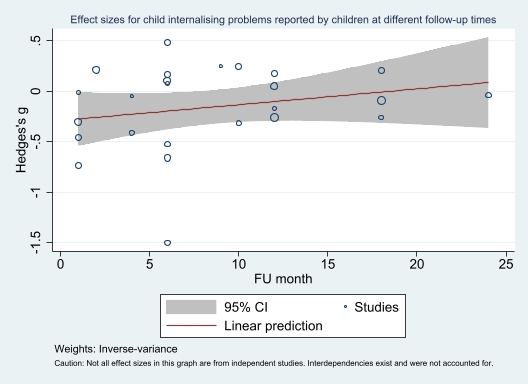


### Effect sizes of parent internalising problems per study and follow-up


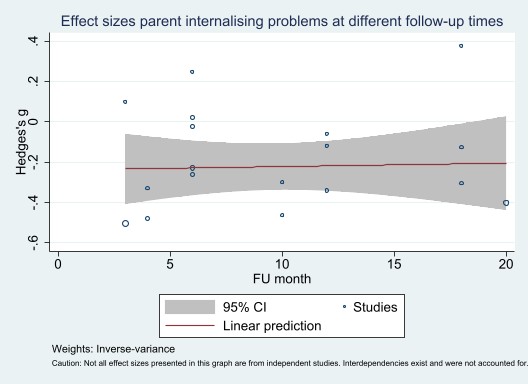


## Bias assessment

### Funnel plots

We conducted multiple funnel plots for studies child and parent mental health outcomes. Additionally, we conducted Egger’s test. All are presented below.


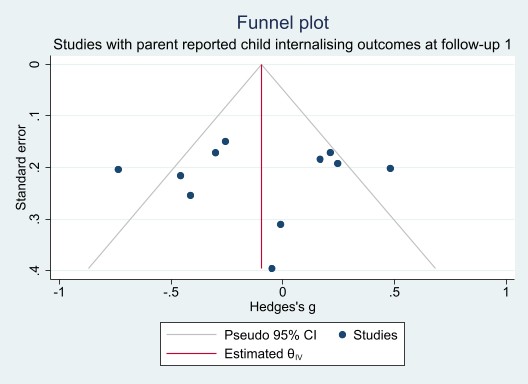


Figure 25S Funnel plot including studies with parent reported child outcomes


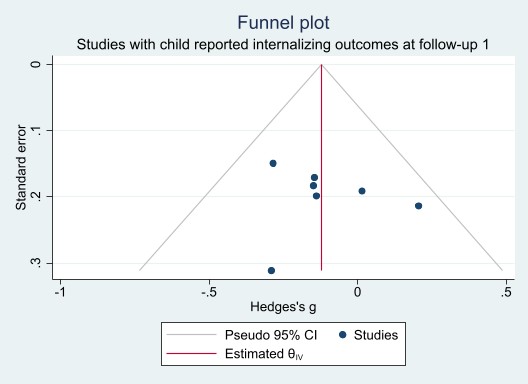


Figure 26S Funnel plot including studies with child reported child outcomes


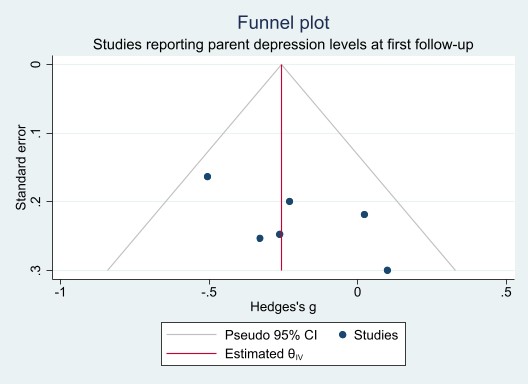


Figure 27S Funnel plot including studies with parent outcomes

### Galbraith plots


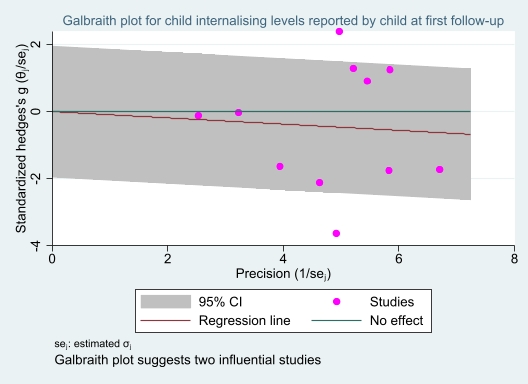


Figure 28S Galbraith plot child internalising levels reported by children at first follow-up


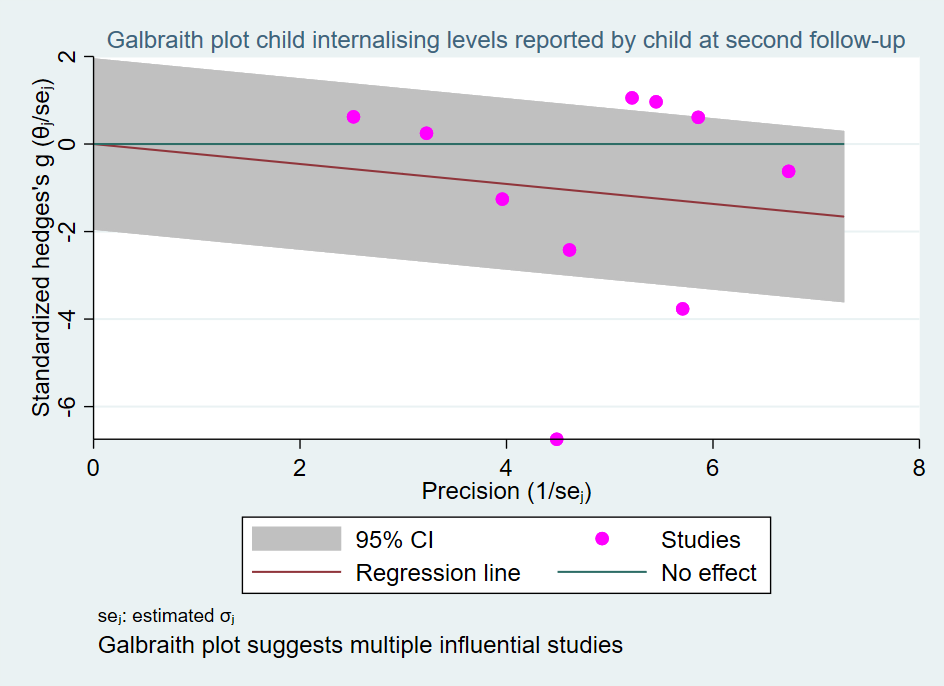


Figure 29S Galbraith plot child internalising levels reported by children at second follow-up


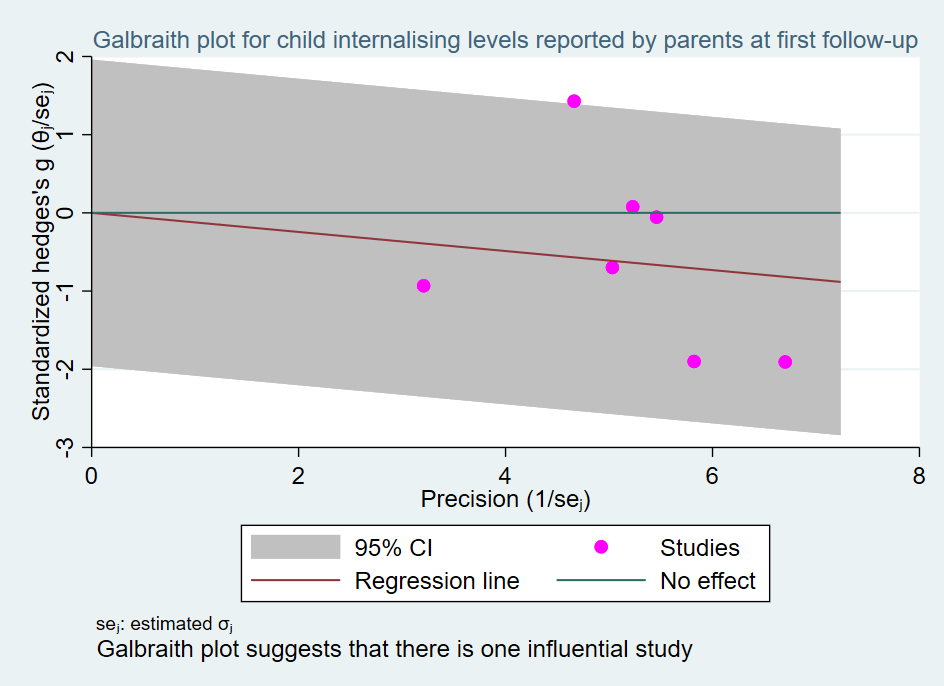


Figure 30S Galbraith plot child internalising levels reported by parents at first follow-up


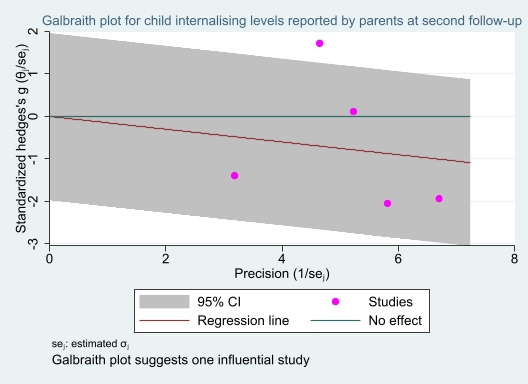


Figure 31S Galbraith plot child internalising levels reported by parents at second follow-up


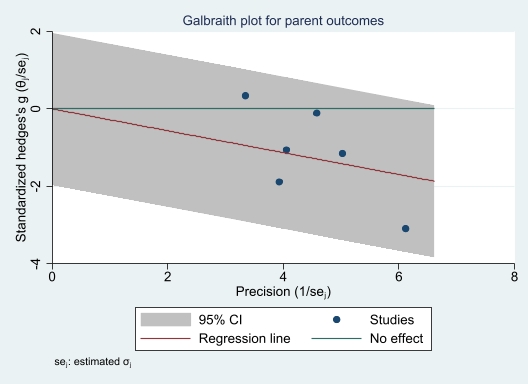


Figure 32S Galbraith plot parent mental health outcomes at first follow-up

## Egger’s tests:

**Child report internalising (A measures)**

Regression-based Egger test for small-study effects

H0: beta1 = 0; no small-study effects

beta1 =1.34

SE of beta1 =2.164

z = 0.62

Prob > |z| = 0.5362

**Child report internalising (B measures)**

Egger test for small-study effects

H0: beta1 = 0; no small-study effects

beta1 = 1.10

SE of beta1 = 1.988

z = 0.55

Prob > |z| = 0.5802

**Parent report child internalising (A measures)**

Regression-based Egger test for small-study effects

H0: beta1 = 0; no small-study effects

beta1 = -0.28

SE of beta1 = 2.019

z = -0.14

Prob > |z| = 0.8889

**Parent report child internalising (B measures)**

Regression-based Egger test for small-study effects

H0: beta1 = 0; no small-study effects

beta1 = 0.75

SE of beta1 = 1.775

z = 0.42

Prob > |z| = 0.6715

**PARENT INTERNALISING**

Regression-based Egger test for small-study effects

H0: beta1 = 0; no small-study effects

beta1 = 3.48

SE of beta1 = 2.114

z = 1.65

Prob > |z| = 0.0996

## Trials excluded from meta-analysis with reasons

| Trial ID | Paper ID | Country | Sample | Control group | Design | Quality rating | Reason for exclusion |
| --- | --- | --- | --- | --- | --- | --- | --- |
| 2 | 73 | Germany | N_t_ 28  N_c_ 49 | passive | fRCT | ** | Pilot study |
| 5 | 180 | USA | N_t+c_ 28 | active | RCT | ** | no descriptive statistics provided |
| 6 | 148  162  177  178  179 | USA | N_t_ 55  N_c_ 44 | active | RCT  RCT  RCT  RCT  RCT | ***  *****  ***  ***  **** | no descriptive statistics provided for mental health outcomes |
| 10 | 10 | UK | N_t_ 20  N_c_ 15 | TAU | MIXED (fRCT) | *** | feasibility trial |
| 11 | 11  19  31  48 | USA | N_t_ 123  N_c_ 66 | active | RCT  RCT  RCT  RCT | *  ***  -  ** | - no mental health measures  - insufficient studies on substance use |
| 17 | 62 | USA | N_t_ 16  N_c_ 8 | active | fRCT | * | Feasibility/pilot trial |
| 20 | 86 | USA | N_t_ 35  N_c_ 37 | TAU | RCT | ***** | - no mental health measures  -insufficient studies on substance use |
| 24 | 118 | Germany | 375^1^ | TAU | NRCT | ** | no descriptive statistics provided |
| 26 | 143 | Spain | N_t_ 15  N_c_ 16 | passive | NRCT | *** | no descriptive statistics provided |
| 28 | 173  176 | USA | N_t_ 77  N_c_ 58 parents | TAU | RCT  RCT | **  *** | no mental health outcomes |
| 31 | 92 | USA | N_t_ 25  N_c_ 18 | TAU | fRCT | ***** | feasibility/pilot trial |
| 32 | 208 | USA | N_t_ 43  N_c_ 44 | TAU | RCT | *** | no mental health outcomes |
| 33 | 6  202 | USA | N_t_ 61 youth  N_c_ 66 youth | active | RCT  RCT | *****  **** | insufficient trials with incidence data |
| 34 | 205  211 | China | N_t_ 37  N_c_ 74 | active | NRCT  NRCT | **  *** | no mental health outcomes |
| 35 | 212 | Iran | N_t_ 30  N_c_ 30 | TAU | NRCT | *** | no post-intervention descriptive statistics |
| 36 | 94 | USA | NC_t_ 21  NC_c_ 19 | active | RCT | ***** | insufficient trials with incidence data |

## 
